# SF3B4 promotes Twist1 expression and clear cell renal cell carcinoma progression by facilitating the export of KLF 16 mRNA from the nucleus to the cytoplasm

**DOI:** 10.1038/s41419-022-05534-w

**Published:** 2023-01-13

**Authors:** Zhan Yang, Ya-Xuan Wang, Jin-Kun Wen, Hai-Tao Gao, Zhen-Wei Han, Jin-Chun Qi, Jun-Fei Gu, Chen-Ming Zhao, Hong Zhang, Bei Shi, Dan-Dan Wang, Xiao-Lu Wang, Chang-Bao Qu

**Affiliations:** 1 Department of Urology, The Second Hospital of Hebei Medical University, 215 Heping W Rd, Shijiazhuang, 050000 China; 2 Molecular Biology Laboratory, Talent and Academic Exchange Center, The Second Hospital of Hebei Medical University, Shijiazhang, China; 3 Department of Biochemistry and Molecular Biology, Ministry of Education of China, Hebei Medical University, No. 361 Zhongshan E Rd, Shijiazhuang, 050017 China

**Keywords:** Oncogenes, Metastasis, Cell migration

## Abstract

Splicing factor 3B subunit 4 (SF3B4) plays important functional roles not only in pre-mRNA splicing, but also in the regulation of transcription, translation, and cell signaling, and its dysregulation contributes to various diseases including Nager syndrome and tumorigenesis. However, the role of SF3B4 and underlying mechanisms in clear cell renal cell carcinoma (ccRCC) remain obscure. In the present study, we found that the expression of SF3B4 was significantly elevated in ccRCC tissues and negatively correlated with the overall survival of ccRCC patients. Upregulation of SF3B4 promotes migration and invasion of ccRCC cells in vitro and in vivo. The promoting effect of SF3B4 on cell migration and invasion is mediated by Twist1, a key transcription factor to mediate EMT. Interestingly, SF3B4, a component of the pre-mRNA spliceosome, is able to promote KLF16 expression by facilitating the transport of KLF16 mRNA into the cytoplasm. Mechanistically, SF3B4 promotes the export of KLF16 mRNA from the nucleus to the cytoplasm and thus enhances KLF16 expression, and in turn elevated KLF16 directly binds to the Twist1 promoter to activate its transcription, leading to EMT and ccRCC progression. Our findings provide evidence that the SF3B4-KLF16-Twist1 axis plays important functional roles in the development and progression of ccRCC, and manipulating this pathway may be a novel therapeutic target for the treatment of ccRCC.

## Facts

1. The expression of SF3B4 is significantly elevated in ccRCC tissues and negatively correlates with the overall survival of ccRCC patients.
2. Upregulation of SF3B4 promotes migration and invasion of ccRCC cells by facilitating Twist1 expression in vitro and in vivo.
3. SF3B4, a component of the pre-mRNA spliceosome, is able to promote KLF16 expression by facilitating the transport of KLF16 mRNA into the cytoplasm.
4. The elevation of KLF16 directly binds to the Twist1 promoter to activate Twist1 transcription.
5. SF3B4-KLF16-Twist1 axis plays important functional roles in the development and progression of ccRCC.

## Open questions

SF3B4, mainly in the nucleus, is a component of the pre-mRNA spliceosome. How does it facilitate the transport of KLF16 mRNA into the cytoplasm?

## Introduction

Renal cell carcinoma (RCC) originates from the malignant transformation of renal tubular epithelial cells and is one of the most common malignant tumors in humans [1]. In 2021, there were about 79,000 new cases of kidney cancer and more than 13,920 deaths in the United States [2]. The incidence of RCC is increasing worldwide and the prognosis remains poor. Clear cell RCC (ccRCC) is the most common histological subtype of RCC, accounting for 75 to 80% of RCC cases [3, 4]. Early-stage or localized RCC is often treated with partial or radical nephrectomy to prolong patient survival [5, 6]. However, more than 25% of patients are diagnosed with metastatic RCC (mRCC), including those who have undergone nephrectomy [7–9]. Although tyrosine kinase inhibitors (CTKI), rapamycin protein (mTOR) inhibitors, and immunotherapy show promise for RCC treatment, most patients still experience cancer progression and eventual death [7, 9, 10]. Recent studies have demonstrated that ccRCC development is closely correlated with epithelial-mesenchymal transition (EMT), which involves the abnormal expression of EMT transcription factors and subsequent downregulation of E-cadherin and upregulation of β-catenin and vimentin. However, little is known about how the expression of EMT transcription factors, including Twist1, is regulated in ccRCC growth and metastasis. Therefore, we urgently need a more accurate understanding of the regulatory mechanisms of EMT transcription factors for better therapeutic strategies.

Splicing factor 3B subunit 4 (SF3B4), a core subunit of the SF3B complex, is a part of the U2-type spliceosome [11]. Recent studies have revealed that SF3B4 plays important roles in the regulation of transcription, translation, and cell signaling in addition to pre-mRNA splicing. Importantly, emerging evidence suggests that the dysregulation of SF3B4 expression by gene mutations or other factors has been implicated in various diseases, including tumorigenesis and Nager syndrome (NS) [12–14]. For example, the upregulation of SF3B4 promotes tumorigenesis in hepatocellular carcinoma (HCC) [12], esophageal squamous cell carcinoma (ESCC) [13], and ovarian cancer (OC) [14], whereas its downregulation or depletion results in pancreatic cancer [15] and breast cancer in NS patient [16]. These findings indicate that SF3B4 plays distinct functional roles in different contexts and different cancer cells, strongly suggesting an essential role of SF3B4 in cell growth and the importance of a finely regulated level of SF3B4 for the regulation of cell homeostasis. Despite the involvement of SF3B4 in different tumors, molecular mechanisms underlying its function in the development and progression of ccRCC remain largely unknown.

Krüppel-like factors (KLFs), which are zinc finger-containing transcription factors, bind to GC-rich DNA regulatory regions of target genes to activate or repress transcription and play key roles in diverse physiological and pathophysiological processes, including proliferation, differentiation, metastasis, inflammation, and pluripotency [17]. In tumorigenesis and development, KLFs can act as tumor suppressors or oncogenes depending on the specific cellular context [18]. Emerging evidence has shown that KLF family members are involved in the development and progression of ccRCC, such as KLF2, KLF4, KLF5, KLF6, KLF8, and KLF9 [19–24]. In addition, several lines of evidence indicate that KLF16 is implicated in a variety of tumor processes, including prostate cancer, breast cancer, bladder cancer, gastric cancer and lung cancer [25–29]. Furthermore, KLF16 overexpression promotes migration and invasion of breast cancer cells by facilitating EMT [27]. However, the potential involvement of KLF16 in ccRCC and the relationship between KLF16 and SF3B4 had not yet been shown.

In this study, we studied the role and mechanisms of SF3B4, which is significantly upregulated in ccRCC tissues, in regulating the migration and invasion of ccRCC cells. Our findings reveal, for the first time, that elevated SF3B4 promotes the export of KLF16 mRNA from the nucleus to the cytoplasm and thus enhances KLF16 expression, and in turn increased KLF16 binds to the Twist1 promoter to activate its transcription, leading to EMT and ccRCC cell migration. Our findings establish a mechanistic link between SF3B4 elevation and ccRCC progression and may provide a novel target for the treatment of ccRCC.

## Materials and methods

### Clinical samples

Patients of ccRCC and corresponding normal kidney tissue were obtained from the Department of Urology, the Second Hospital of Hebei Medical University, Shijiazhuang, China. There were 53 patients in total, including 39 men and 14 women. The average age of patients was 63 years. In the pT status phase, 31 patients were pT1–pT2, and 22 were pT3–pT4. In the pN status phase, there were 30 patients with pN0 and 23 with pN1–pN3. In the TNM phase, there were 33 patients with I–II and 20 with III–IV. In the Furhman class, 29 patients were G1/G2 and 24 were G3/G4. Clinical tissues were collected and frozen in liquid nitrogen, followed by storage at −80 °C. Each participant patient provided written informed consent, and the protocol was approved by the Ethics Committee of Hebei Medical University’s Second Hospital.

### Cell lines and treatment

We obtained human ccRCC cell lines 769-P, SW839, A498, Caki-1, and 786-0 from ATCC (Maryland, USA) and stored them in our laboratory. The 293 A cell line is preserved in our laboratory. Above cells were cultured in DMEM (Gibco, low sugar) supplemented with 10% fetal bovine serum (Clark Bio, Claymont, DE, USA) and 1% penicillin/streptomycin (Solarbio, Beijing) and maintained in a humidified environment with 95% air and 5% CO_2_. To knock down specific genes, such as SF3B4 and KLF16, we used pLKO vectors to induce shRNA. The pLKO vector is a third-generation lentiviral plasmid containing puromycin selection. We utilized pWPI (Addgene, #12254), a second-generation clostridial lentiviral vector that allows simultaneous expression of transgenes and EGFP markers, overexpressing specific genes. Lentiviral pLKO-SF3B4 (shSF3B4), pLKO-KLF16 (shKLF16), pWPI-SF3B4 (SF3B4 expression plasmid, oeSF3B4), pWPI-Twist1 (oeTwist1), pWPI-KLF16 (oeKLF16) and plasmids were designed and constructed from Biocaring Biotechnology Co., Ltd (Shijiazhuang) [3, 30]. The siRNAs for Transcription factors were purchased from GenePharma (Shanghai, China). Lipofectamine 2000 (Thermofisher, #11668019) was used for cell transfection according to the manufacturer’s manual and previous description. Briefly [3], Caki-1 and 769-P cells (1 × 10^5^ cells/ml) were seeded into plates in growth medium (DMEM, Gibco). Until the required number of cells (80% confluence) is obtained at the time of transfection. The cells were washed twice with PBS and the transfected Lipofectamine was added to each well. It was mixed gently by rocking the plate back and forth. The cells were cultured in DMEM (without FBS), 5% CO_2_, and 37 °C humidified incubator for 4–6 h. Then the cells were washed twice with PBS, replaced with complete medium, and cultured for 48 h.

### RNA isolation and qRT-PCR analysis

Total RNA Kit II (Omega, #R6934) was used to isolate total RNA from tissues and cultured cells according to the manufacturer’s manual. The NanoDrop 2000 spectrophotometer was used to measure RNA concentration and quality. The cDNA first strand was synthesized by using MonScript™ RTIII Super Mix with dsDNase (Mona, #MR05201M) with oligo (dT) primer. A qRT-PCR analysis on mRNA was performed using Platinum SYBR Green qPCR Super Mix UDG kit (Invitrogen, # 11733046) on an ABI 7500 FAST System using diluted cDNA. GAPDH was used as a reference gene to standardize gene transcriptional expression. The calculation was performed using the 2^-ddCt^ formula as described earlier [31]. All used primers in this study were summarized in Supplementary Table 2.

### RNA sequencing (RNA-seq)

Total RNAs were extracted from three groups of pWPI and shSF3B4 transfected-Caki-1 by using Total RNA Kit II (Omega, #R6934) and then removing rRNAs with the RiboMinus Eukaryote Kit (Qiagen, Valencia, CA) before constructing the library. Next, the Illumina HiSeq 2000 was used to the sequenced RNA-seq library. Sequencing reads of RNA were aligned to the human genome using the software STAR and RNA abundance quantification was performed using the software RSEM.

### Western blotting

The protein was extracted from the cultured cells and frozen tissue samples using RIPA lysis buffer and protease inhibitor cocktail as described previously [30]. The same amount of protein was loaded onto the gel using the modified Bradford method for protein quantitative analysis. After being separated by SDS-PAGE, the proteins were electrotransferred to a polyvinylidene fluoride membrane (Millipore, IPVH00010). The membrane was blocked with 5% nonfat milk for 2 h. Finally, the membranes were incubated with the primary antibody overnight at 4 °C. The antibodies used were as follows: SF3B4 (Proteintech, 1:1000, 10482-1-AP), MMP1 (Proteintech, 1:1000, 10371-2-AP), E-cadherin (Proteintech, 1:500, 20874-1-AP), vimentin (Proteintech, 1:1000, 10366-1-AP), Twist1 (Proteintech, 1:1000, 25465-1-AP), ZO-1 (Proteintech, 1:500, 21773-1-AP), KLF16 (Abcam, 1:500, ab187973) and β-actin (Cellsignal, 1:1000, sc-47778). After reaction with HRP-labeled secondary antibody (1:10000, Rockland), the membrane was treated with Immobilon™ Western chemiluminescence HRP substrate (Millipore) and detected by ECL (enhanced chemiluminescence) Fuazon Fx (Vilber Lourmat).

### Hematoxylin and eosin, immunohistochemistry, and immunofluorescence staining

Fresh ccRCC and normal kidney tissue are fixed in formalin and sliced 4-μm thick. Tissue sections were used for hematoxylin and eosin, immunohistochemistry, and immunofluorescence staining. For immunohistochemical staining analyses [32], tissue sections were decompressed and immersed in water before performing antigen retrieval. After treatment with H_2_O_2_, slides were blocked by fetal bovine serum for 30 min. Then, the sections were incubated with primary antibodies overnight at 4 °C. HRP‐conjugated secondary antibodies was applied to the slides, and DAB was used to visualize. For immunofluorescent staining [30], cells and tissue slides were permeabilized with 0.5% Triton X‐100, then blocked with 5% goat serum, and incubated with primary antibody. Fluorescein-labeled secondary antibodies were reacted with slides and DAPI was used to stain the nuclei. The cross-sectional image was acquired with a Leica microscope (Leica DM6000B, Switzerland) and digitized with LAS V.4.4 (Leica).

### Immunofluorescence combined with fluorescence in situ hybridization

Cell slides were fixed in 4% paraformaldehyde and performed in situ hybridization according to the user manual of miRCURY LNATM microRNA ISH Optimization Kit (Exiqon) as previous description [33]. Hybridization was performed using fluorescence-labeled KLF16 mRNA probes in hybridization buffer (Exiqon). After stringent washing with SSC buffer, slides were blocked with 10% normal goat serum. The sections were then incubated with anti-SF3B4 primary antibody (Ptoteintech, 10482-1-AP) for 1 h. After washing, the slides were incubated with a rhodamine-labeled secondary antibody (KPL, USA, 031506). Images were acquired using a Leica microscope (Leica DM6000B, Switzerland) and digitized with software of LAS V.4.4 (Leica).

### ChIP assay

Caki-2 cells were treated with formaldehyde for ChIP assay as previously described [3]. The cross-linked chromatin was sonicated to an average size of 400–600 nt. Then the samples were diluted 10-fold and incubated with protein A-agarose/salmon sperm DNA. The DNA fragments were immunoprecipitated overnight at 4 °C with anti-KLF16 or anti-IgG antibodies. After the reversal of cross-linking, KLF16 occupancy on the Twist1 promoter was detected by qRT-PCR. The ChIP primer sequences are summarized in Additional file 2.

### Transwell and 3D Matrigel drop invasion assays

Transwell assay was performed as previous description [30]. After indicated treatment, cells were seeded in the top chamber of the insert (Corning, 3422) and were allowed to invade through with or without Matrigel (BD, #356234) blocking. 24 h later, cells on the lower surface were fixed in 100% methanol and stained with 0.05% crystal violet. Cells were performed 3D Matrigel drop invasion assay as described previously [34] 769-P or Caki-1 cells were transfected as indicated and then suspended in 10 μl Matrigel. A mixture contains 5 × 10^4^ cells and Matrigel was pipetted as a droplet into a 12-well plate for 15 minutes to form Matrigel drop prior to adding media. After 7 days of culture, the radial distance of the cell had migrated away from the edge of tumoroids was measured.

### Xenograft animal model

The generation of the xenograft model is as described previously [30, 32, 33]. Briefly, male BALB/c nude mice aged 4–6 weeks were purchased from Vital River Laboratory Animal Technology Co., Ltd. (Beijing, China). A total of 5 × 10^6^ stable shSF3B4 or oeKLF16-infected Caki-2 cells were collected by trypsinization and resuspended in 0.2 mL PBS mixed with 50% Matrigel (BD, #356234); the suspension was injected subcutaneously to the right back side. Measure the length and width of mouse tumors with calipers twice a week. Then, we use the following formula to calculate the tumor volume: tumor volume = (length × width 2)/2. At the end of the experiment, the mice were euthanized and samples were collected for further detection.

### Luciferase assays

Twist1 2000 bp promoter was amplified and inserted into pGL3-basic plasmid and then Sanger sequencing for confirming. Caiki-1 cells were seeded in 24-well plate and co-transfected with indicated vectors. Luciferase activity was measured as the previous description [35] by Dual-Glo Luciferase Assay System (Promega) with a Flash and Glow reader (LB955; Germany). The specific target activity was expressed as the relative activity ratio of firefly luciferase to Renilla luciferase.

### Bioinformatics Analysis

In the TCGA-KIRC database, mRNA expression levels of genes in ccRCC patients and clinical characteristics were obtained from UCSC XENA (https://xenabrowser.net/datapages/). This dataset consists of 533 ccRCC tissues and 72 normal kidney tissues. The gene expression profiling dataset (GSE53757) were obtained from Gene Expression Omnibus (GEO) database (https://www.ncbi.nlm.nih.gov/gds).

### Statistical analysis

Data were presented as the mean ± SEM. Student’s *t* test was used to analyze the differences between the two groups. Spearman’s correlation analysis was used to evaluate the correlation analysis. Values of *P* < 0.05 were considered statistically significant. GraphPad Prism 8.0 software was used for the statistical analysis (GraphPad Software).

## Results

### SF3B4 is significantly upregulated in ccRCC tissues and negatively correlated with the survival of ccRCC patients

**Fig. 1:**
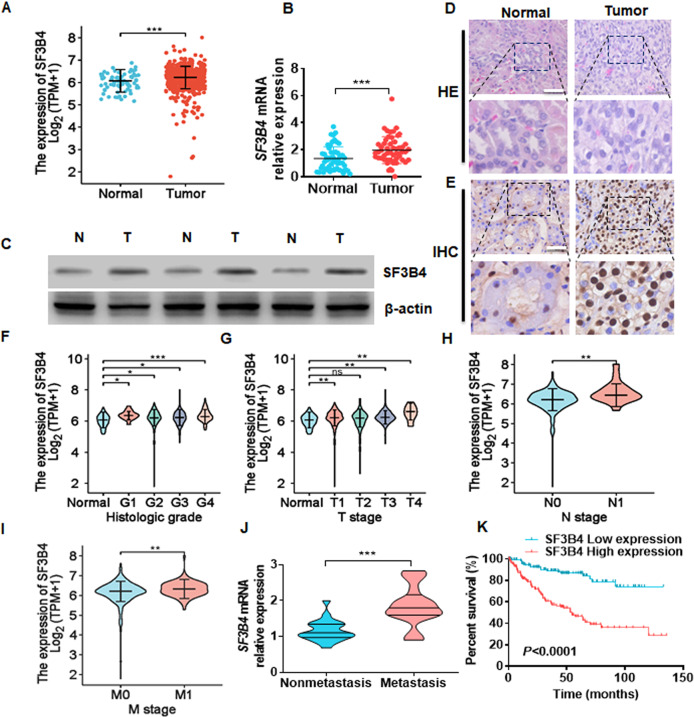
SF3B4 is upregulated and negatively correlates with survival in patients with ccRCC. **A** The expression of SF3B4 was downloaded and then analyzed from the TCGA database in normal (*n* = 72) and ccRCC (*n* = 533) tissues. **B** The mRNA levels of SF3B4 were detected by RT-qPCR in total 53 clinical samples. **C** Three groups of ccRCC and normal renal tissue were randomly selected and western blot was used to detect SF3B4 protein expression. **D** Hematoxylin and eosin (HE) staining of tumor and normal kidney tissues. Scale bar = 50 μm. **E** Immunohistochemical staining examined the SF3B4 expression in clinical samples. Scale bar = 50 μm. **F**–**I** The mRNA levels of SF3B4 were compared with different clinicopathological parameters from TCGA‐KIRC: cancer versus normal tissues, G stage, T stage, N stage, and M stage. **j** RT-qPCR detected the SF3B4 expression in the ccRCC tissues of metastasis (n = 21) and non-metastasis (*n* = 21). **K** Poor prognosis and low overall survival were associated with high SF3B4 expression in TCGA‐KIRC. All data are expressed as the mean ± SEM of three independent experiments. *P < 0.05, **P < 0.01, ***P < 0.001 vs. their corresponding controls.

To explore whether SF3B4 is involved in the development of ccRCC, we first analyzed its mRNA level in the samples in the pan-cancer database of TCGA and found that SF3B4 was universally upregulated in a variety of tumors, including ccRCC (Supplementary Fig. 1; Fig. 1A). Next, we examined SF3B4 expression in 53 pairs of ccRCC tissues and their adjacent non-carcinoma tissues and confirmed a significant increase in SF3B4 mRNA and protein levels in ccRCC tissues (Fig. 1B, C). Histomorphological and immunohistochemical analyses yielded the same results (Fig. 1D, E). In addition, we evaluated the relationship between SF3B4 expression and clinicopathological features (Table 1). A higher expression level of SF3B4 was associated with higher histologic grade, higher pathologic stage, clinical N stage, and M stage in ccRCC patients (Fig. 1F–I). Furthermore, SF3B4 expression was higher in patients with metastasis than in patients without metastasis (Fig. 1J). Importantly, the high level of SF3B4 expression in ccRCC patients was associated with poor overall survival (OS, *P* < 0.0001) (Fig. 1K). Together, these results suggest that SF3B4 plays an important role in the carcinogenesis and progression of ccRCC.

**Table 1:** Clinicopathological characteristics.

Characteristics	Number of patients (*n*)	SF3B4 expression		
		Low (%)	High (%)	*P* value
No. of patients	53	27	26	
Age				
≤63	27	13 (48.15)	14 (51.85)	0.678
>63	26	14 (53.85)	12 (46.15)	
Gender				
Male	39	20 (51.28)	19 (48.72)	0.934
Female	14	7 (50.00)	7 (50.00)	
Tumor size (cm)				
≤5	35	16 (45.71)	19 (54.29)	0.288
>5	18	11 (61.11)	7 (38.89)	
pT status				
pT_1_–pT_2_	31	12 (38.71)	19 (61.29)	0.034
pT_3_–pT_4_	22	15 (68.18)	7 (31.82)	
pN status				
pN0	30	11 (36.67)	19 (63.33)	0.018
pN1–pN3	23	16 (69.57)	7 (30.43)	
TNM stage				
I–II	33	13 (39.39)	20 (60.61)	0.031
III–IV	20	14 (70.00)	6 (30.00)	
Furhman Grade				
G1/G2	29	10 (34.48)	19 (65.52)	0.008
G3/G4	24	17 (70.83)	7 (29.17)	

### The upregulation of SF3B4 promotes migration and invasion of ccRCC cells

**Fig. 2:**
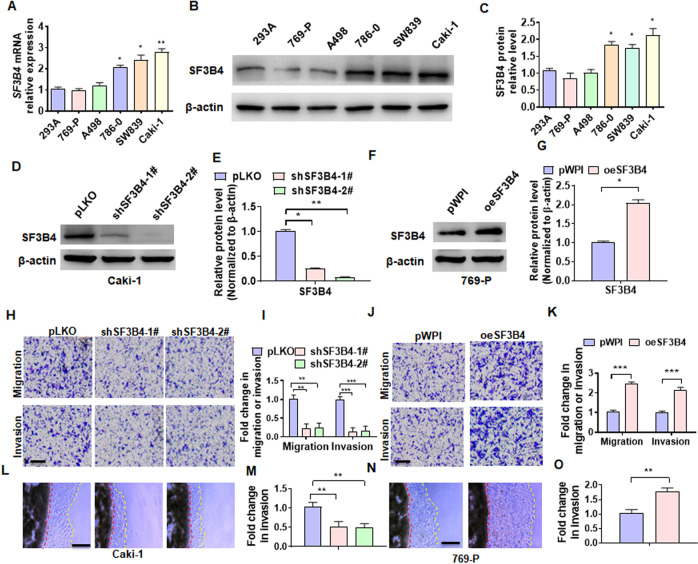
The upregulation of SF3B4 promotes migration and invasion of ccRCC cells. **A**, **B** RT-qPCR and Western blotting detected the SF3B4 expression in different cell lines of ccRCC. **C** Quantitative analysis of **B**. **D**, **E** Caki-1 cells were transfected with pLKO, shSF3B4-1#, or shSF3B4-2#, and then western blot analysis examined the SF3B4 and MMP1 expression. **F**, **G** 769-P cells were transfected with pWPI or pWPI-SF3B4 (oeSF3B4) and then SF3B4 and MMP1 were examined by western blot. **H** Caki-1 cells were treated as in **D** and transwell assay and detected the migration and invasion of cells. Scale bar = 50 μm. **I** Quantitative analysis of **H**. **J** 769-P cells was treated as in **F** and then the migration and invasion of cells were detected by transwell assay. **K** Quantitative analysis of **J**. **L** Caki-1 cells were treated as in **D** and a 3D Matrigel drop invasion assay detected the invasion of cells. Scale bar = 100 μm. **M** Quantitative analysis of **L**. **N** 769-P cells were treated as in **F** and a 3D Matrigel drop invasion assay detected the invasion of cells. **O** Quantitative analysis of **N**. All data are expressed as the mean ± SEM of three independent experiments. *P < 0.05, **P < 0.01, ***P < 0.001 vs. their corresponding controls.

To clarify the functional roles of SF3B4 in ccRCC development and progression, we first detected the SF3B4 expression in different RCC cell lines, and found that the SF3B4 expression was higher in Caki-1 cell but lower in 769-P cell (Fig. 2A–C). In the following experiments, we knocked down SF3B4 in Caki-1 cells and overexpressed it in769-P cells. As shown in Fig. 2D, E, and Supplementary Fig. 2A, transfection of two shRNAs produced a reduction of more than 70% of SF3B4 protein or mRNA level in Caki-1 cells. Conversely, overexpression of SF3B4 in 769-P cells markedly elevated SF3B4 protein and RNA levels (Fig. 2F, G and Supplementary Fig. 1B). We then investigated whether SF3B4 promotes migration and invasion of ccRCC cells in vitro. A transwell assay was used to examine the cell migration and invasion and the results showed that depletion of SF3B4 in Caki-1 cells inhibited, while its overexpression in 769-P cells promoted the migration and invasion (Fig. 2H–K). Furthermore, using a 3D Matrigel droplet assay, we also found that the radial distance of Caki-1 cells migrating from the edge of the Matrigel droplet in SF3B4-depleted cells was much shorter than in the control group (Fig. 2L, M). In contrast, 769-P cells overexpressing SF3B4 exhibited longer migration distances (Fig. 2N, O). These findings establish a direct relationship between SF3B4 upregulation and cell migration and invasion.

### Twist1 mediates SF3B4-induced EMT in ccRCC

**Fig. 3:**
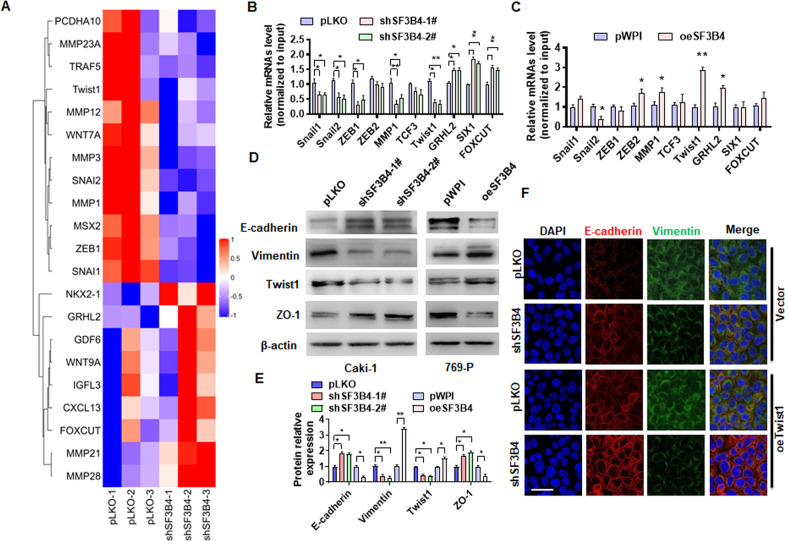
Twist1 mediates SF3B4-induced EMT in ccRCC. **A** Gene expression profiles in pLKO or shSF3B4-transfected Caki-1 cells. Heat map of hierarchical clustering indicates the differentially expressed genes (red: upregulation; blue: downregulation). **B**, **C** Caki-1 (**B**) and 769-P (**C**) cells were transfected with shSF3B4 or oeSF3B4, and then RT-qPCR detected the expression of the indicated genes. **D** The expressions of EMT-related genes, E-cadherin, vimentin, Twist1, and ZO-1 were detected by western blot in shSF3B4-transfected Caki-1 or oeSF3B4-transfected 769-P cells. **E** Quantitative analysis of **D**. **F** Caki cells were transfected with shSF3B4 or oeTeist1 or control vector, alone or together, and the expression of E-cadherin and vimentin were detected by double immunofluorescence staining. Scale bar = 20 μm. All data are expressed as the mean ± SEM of three independent experiments. *P < 0.05, **P < 0.01 vs. their corresponding controls.

To determine the downstream effector of SF3B4 that mediates SF3B4-induced ccRCC cell migration, we first knocked down SF3B4 in Caki-1 cells and confirmed SF3B4 and EMT relative gene expression (Supplementary Fig. 3A). And then high-throughput transcriptome sequencing was used to identify genes that are regulated by SF3B4. The results showed that expressions of many invasion and migration-related genes in Caki-1 cells, such as MMP1, Twist1, Snail1, Snail2, and ZEB1 were obviously downregulated upon the deletion of SF3B4 (Fig. 3A). Since SF3B4 is involved in regulating migration of ccRCC cells, we will further validate these metastasis-related genes in RT-qPCR (Fig. 3B). Subsequently, we overexpressed SF3B4 in 796-P cells and found that reduced expression of MMP1 and Twist1 was reversed by overexpressing SF3B4 (Fig. 3C). Especially, Twist1, a key transcription factor to mediate EMT, was markedly downregulated in the SF3B4-deficient Caki-1 cells, but upregulated in SF3B4-overexpressed 796-P cells (Fig. 3B, C). We examined EMT-related gene expression in different cell lines and found that Vimentin has a relative low expression in 769-P cell and high expression in Caki-1 cells. Conversely, the protein of E-cadherin with a relative high level in 769-P cells and a low level in Caki-1 cells (Supplementary Fig. 3B). Further, Western blot analysis showed that knockdown of SF3B4 in Caki-1 cells reduced the expression of mesenchymal markers vimentin and Twist1, while increased the expression of the epithelial markers E-cadherin and ZO-1. The opposite results were observed in SF3B4-overexpressing 796-P cells (Fig. 3D, E). Furthermore, immunofluorescence staining yielded similar results (Fig. 3F). The experiments described above establish a mechanistic link between SF3B4 elevation and EMT as well as subsequent ccRCC progression.

### Twist1 mediates SF3B4-induced cell migration and its upregulation contributes to the progression of ccRCC

**Fig. 4:**
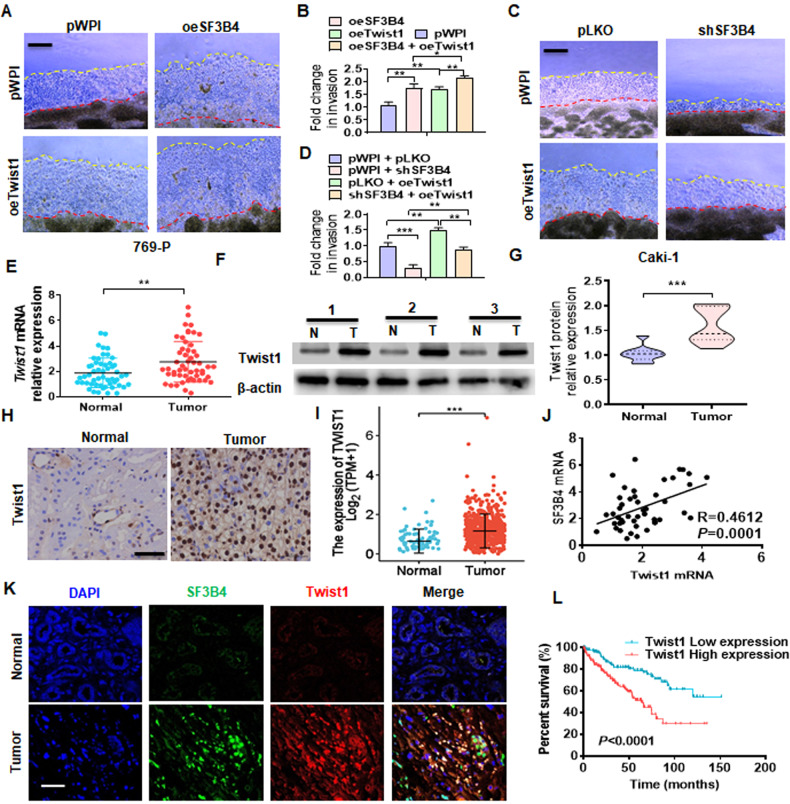
Upregulation of Twist1 contributes to the progression of ccRCC. **A** 769-P cells were transfected with oeSF3B4 or oeTwist1, alone or together, and then a 3D Matrigel drop invasion assay detected the invasion of cells. Scale bar = 100 μm. **B** Quantitative analysis of **A**. **C** Caki-1 cells were transfected with shSF3B4 or oeTwist1, alone or together, and then a 3D Matrigel drop invasion assay detected the invasion of cells. **D** Quantitative analysis of **C**. **E** RT-qPCR detected the Twist1 mRNA expression in normal (*n* = 53) and ccRCC (*n* = 53) tissues. **F**, **G** Twist1 protein level in normal or ccRCC tissues was measured by western blot. **H** Immunohistochemical staining detected the Twist1 in clinical samples. Scale bar = 50 μm. **I** Twist1 mRNA level was analyzed from the TCGA database in normal (N, *n* = 72) and ccRCC (T, *n* = 533) tissues. **J** The correlation between SF3B4 and Twist1 mRNA in ccRCC tissues was analyzed by Pearson correlation analysis of our clinical data (R = 0.4612, *P* = 0.0001). **K** Immunofluorescence staining detected the expression of SF3B4 and Twist1 in ccRCC and normal renal tissue. Scale bar = 100 μm. **L** According to the TCGA database with low and high Twist1 levels, the overall survival of ccRCC patients was analyzed using the Kaplan–Meier method. All data are expressed as the mean ± SEM of three independent experiments. **P < 0.01, ***P < 0.001 vs. their corresponding controls.

To further investigate whether Twist1 is responsible for SF3B4-induced cell migration, we examined the effect of Twist1 gain- and loss-of-function on ccRCC cell migration and invasion by using the 3D Matrigel droplet assay. As shown in Fig. 4A, B, transfection with Twist1 plasmid (oeTwist1) markedly promoted the invasion of 769-P cells, and cotransfection with oeTwist1 and SF3B4 plasmids (oeSF3B4) into 769-P cells further enhanced cell migration and invasion compared with transfection with oeTwist1 or oeSF3B4 alone. In contrast, overexpression of Twist1 in Caki-1 cells significantly reversed the inhibitory effect of shSF3B4 on cell invasion (Fig. 4C, D). Next, we measured the Twist1 expression in ccRCC tissues and their adjacent non-carcinoma tissues, and the results showed that the mRNA and protein levels of Twist1 significantly increased in ccRCC tissues compared to normal control tissues (Fig. 4E–G). Immunohistochemical staining and analysis of TCGA database yielded the same results (Fig. 4H, I). In addition, we also found that the expression of Twist1 in metastatic tissues was significantly upregulated (Supplementary Fig. 4). Moreover, the Pearson correlation analysis revealed a positive correlation between SF3B4 and Twist1 mRNA from our clinical data (Fig. 4J). Double immunofluorescence staining confirmed that the high expression of SF3B4 in ccRCC tissues was accompanied by the high expression of Twist1 (Fig. 4K). Notably, the Kaplan-Meier analysis showed that high expression of Twist1 in ccRCC patients predicted poorer overall survival (Fig. 4L). Taken together, these data support that Twist1 mediates SF3B4-induced cell migration and invasion, and its upregulation contributes to the progression of ccRCC.

### KLF16 mediates SF3B4 upregulation of Twist1 expression

**Fig. 5:**
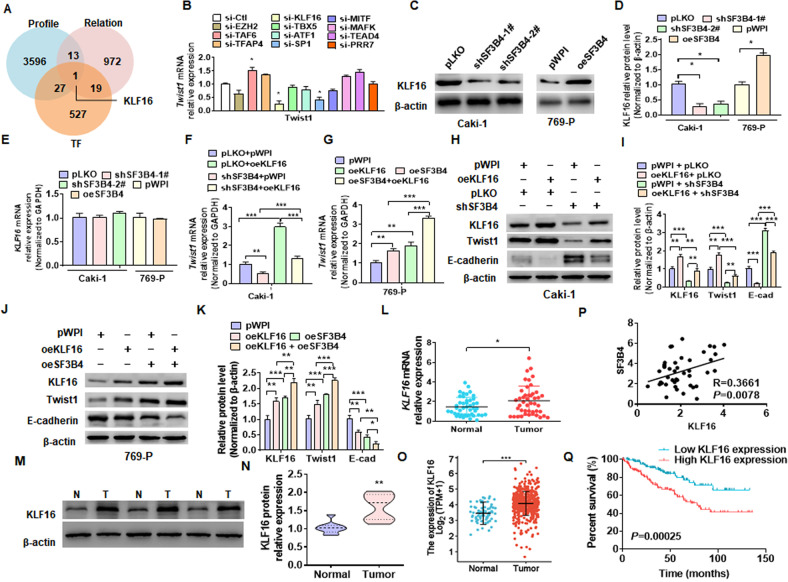
KLF16 mediates SF3B4 upregulation of Twist1 expression. **A** Potential target genes of SF3B4 were analyzed from the data of gene transcriptome of SF3B4-depleted cells (Profile), genes expressed in association with SF3B4 in ccRCC tissues in TCGA data (Relation) and transcription factor (TF), and are shown by a Venn diagram. **B** RT-qPCR detected the Twist1 mRNA expression in Caki-1 cells transfected with the indicated siRNAs for candidate transcription factors. **C**, **D** Western blot examined the KLF16 expression in Caki-1 and 769-P cells transfected with the indicated constructs. **E** Cells were transfected as in **C** and then RT-qPCR detected the KLF16 mRNA expression. **F**, **G** Caki-1, and 769-P cells were transfected as indicated, and then RT-qPCR detected the expression of Twist1 mRNA. **H**, **I** Caki-1 cells were transfected with pWPI-KLF16 (oeKLF16) and shSF3B4, alone or together, and then the protein levels of KLF16, Twist1, and E-cadherin were examined by western blot. **J**, **K** 769-P cells were transfected with oeKLF16 and oeSF3B4, alone or together, and then KLF16, Twist1, and E-cadherin were examined by western blot. **L** KLF16 mRNA expression was detected by RT-qPCR in normal (N, *n* = 53) or ccRCC (T, *n* = 53) tissues. **M** KLF16 protein level was examined by western blot from three pair of randomly selected clinical ccRCC samples. **N** Quantitative analysis of **M**. **O** The expression level of KLF16 was downloaded and then analyzed from data of the TCGA database in normal (*n* = 72) and ccRCC (*n* = 533) tissues. **P** The correlation between SF3B4 and KLF16 mRNA level in ccRCC tissues was analyzed by Pearson correlation analysis of our clinical data (*R* = 0.3661, *P* = 0.0078). **Q** According to the TCGA database, the overall survival for ccRCC patients with low and high Twist1 levels were analyzed by using the Kaplan–Meier method. All data are expressed as the mean ± SEM of three independent experiments. *P < 0.05, **P < 0.01, ***P < 0.001 vs. their corresponding controls.

To investigate how SF3B4, a pre-mRNA splicing factor, promotes the upregulation of Twist1 mRNA and protein, we knocked down SF3B4 and performed the transcriptome analysis. We then attempted to analyze this transcriptome sequencing dataset (Supplementary Table 2) by combining the number of co-expression genes with SF3B4 in renal cancer (Supplementary Table 3) and transcription factors from Uniprot database (Supplementary table 4). We found that transcription factors, KLF16, may be involved in the regulation of SF3B4 to Twist1 (Fig. 5A). Therefore, we knocked down some transcription factors, which were speculated to be related to transcription activation of Twist1 gene, and detected Twist1 mRNA expression in Caki-1 cells. As shown in Fig. 5B, depletion of KLF16 or SP1 significantly reduced Twist1 expression, while siRNA-mediated knockdown of TAF6 elevated Twist1 mRNA level. Subsequently, we knocked down or overexpressed SF3B4 and detected the KLF16 expression. The results showed that the depletion of SF3B4 in Caki-1 cells reduced, while its overexpression in 769-P cells enhanced KLF16 expression (Fig. 5C, D). Interestingly, manipulating the expression of SF3B4 did not affect the mRNA level of KLF16 (Fig. 5E). However, overexpression of KLF16 alone promoted Twist1 expression and completely abolished the inhibitory effect of shSF3B4 transfection on Twist1 expression (Fig. 5F). In parallel, overexpression of KLF16 significantly enhanced SF3B4 overexpression-induced Twist1 upregulation (Fig. 5G). We obtained the same results at the protein level and demonstrated that KLF16 is involved in SF3B4 regulation of Twist1 expression (Fig. 5H–K). In addition, To confirm whether KLF16 mediated SF3B4-regulated Twist1 expression, Caki-1, and 769-P cells were transfected with oeSF3B4 or shKLF16, alone or both together, and then RT-qPCR detected Twist1 expression. As shown in Supplementary Fig. 5A, knocking down KLF16 blocks the promotion effect of SF3B4 on Twist1. Notably, the depletion of KLF16 almost neutralizes the promotion of cell migration by overexpression of SF3B4 (Supplementary Fig. 5B, C). Moreover, The expression of KLF16 was significantly upregulated in ccRCC tissues vs. adjacent non-carcinoma tissues (Fig. 5L, O). We found that KLF16 expression was significantly elevated in metastatic tissues compared to non-metastatic tissues (Supplementary Fig. 5D). The upregulation of KLF16 had a positive correlation with SF3B4 mRNA level in the ccRCC tissues (Fig. 5P), with high expression of KLF16 in ccRCC patients having a significantly poorer survival outcome (Fig. 5Q). These data demonstrate that KLF16 is a key factor that mediates SF3B4 promotion of Twist1 expression.

### SF3B4 upregulates KLF16 expression by promoting its mRNA export from the nucleus

**Fig. 6:**
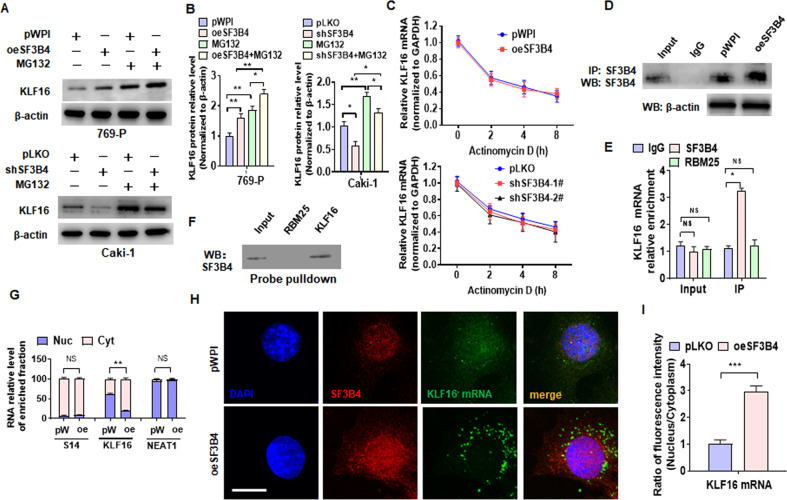
SF3B4 upregulates KLF16 expression by promoting its mRNA export from the nucleus. **A**, **B** Western blot analysis examined KLF16 expression in MG132-treated cells after oeSF3B4 or shSF3B4 transfection. **C** The KLF16 mRNA was examined by RT-qPCR in actinomycin D-treated cells after transfection with the indicated constructs. **D** Caki-1 cells were transfected with oeSF3B4 or empty vector, and SF3B4 in the anti-SF3B4 immunoprecipitates was measured by Western blotting. **E** RNA binding protein immunoprecipitation assay (RIP) detected KLF16 mRNA enrichment by SF3B4 or RBM25 antibody. **F** Biotinylated RNA probes of KLF16 mRNA were used to pull down the RNA-protein complex, and the Western blot analysis detected SF3B4 enrichment. **G** Caki-1 cells were transfected with oeSF3B4 (oe) or pWPI (pW) and then RNA was isolated from the nucleus (Nuc) and cytoplasm (Cyt), mRNA of S14, KLF16, and NEAT RNA was detected by RT-qPCR. S14 acts as plasma-specific RNA and NEAT1 RNA as nucleus-specific RNA. **H** Caki-1 cells were treated as in **G**, and the expression of SF3B4 protein and KLF16 mRNA as well as their distribution in the nucleus and cytoplasm were detected by immunofluorescence combined with FISH. SF3B4 antibody (red) was used to detect SF3B4 protein, while the FITC-probe (green) was used to detect KLF16 mRNA. **I** Quantitative analysis of the KLF16 mRNA distribution in the nucleus and cytoplasm. Scale bar = 5 μm. All data are expressed as the mean ± SEM of three independent experiments. *P < 0.05, **P < 0.01, ***P < 0.001 vs. their corresponding controls.

Because SF3B4 gain- and loss-of-function failed to affect the mRNA level of KLF16, we hypothesized that SF3B4 could upregulate KLF16 expression by inhibiting its ubiquitination. To test this hypothesis, we overexpressed or knocked down SF3B4 in cells and then treated with proteasome inhibitor MG132, and the results showed that MG132 treatment significantly increased KLF16 protein level regardless of transfection with oeSF3B4 or shSF3B4 (Fig. 6A, B), implying that SF3B4-regulated KLF16 does not undergo ubiquitin-mediated degradation in ccRCC cells. Next, we examined the effect of SF3B4 knockdown or overexpression on KLF16 mRNA stability and proved that SF3B4 did not regulate KLF16 mRNA levels by stabilizing mRNA (Fig. 6C). Given that SF3B4 acts as a pre-mRNA splicing factor and RNA binding protein, we hypothesized that SF3B4 could play a role in mRNA export from the nucleus. To test this hypothesis, we first overexpressed SF3B4 in Caki-1 cells and then confirmed through CoIP experiments that SF3B4 molecules can be efficiently pulled down by an SF3B4 antibody (Fig. 6D). Next, we examined the SF3B4 interaction with KLF16 mRNA by RNA binding protein immunoprecipitation (RIP). As shown in Fig. 6E, the mRNA of KLF16 could be efficiently pulled down by the anti-SF3B4 antibody, but not the anti-RBM25 antibody. Consistently, SF3B4 protein was pulled down by using the biotinylated probe of KLF16 mRNA, but not the RBM25 mRNA (Fig. 6F). Notably, we isolated cytoplasmic and nuclear RNA and examined the distribution of KLF16 mRNA. The results showed that KLF16 mRNA was distributed in both the cytoplasm and nucleus under normal conditions, but after overexpression of SF3B4, the distribution of KLF16 mRNA in the cytoplasm was significantly increased, while the distribution in the nucleus decreased. However, the distribution of S14 mRNA and NEAT1 RNA did not change significantly after overexpression of SF3B4 (Fig. 6G). Consistent with this, we performed an immunofluorescence staining combined with FISH by using the SF3B4 antibody and FITC-labeled probe of KLF16 mRNA and confirmed that overexpression of SF3B4 significantly increased KLF16 mRNA, but not NEAT1 RNA, distribution in the cytoplasm (Fig. 6H, I and Supplementary Fig. 6). These findings suggest that SF3B4 upregulates KLF16 expression by exporting RNA from the nucleus to the cytoplasm.

### Upregulation of Twist1 by KLF16 mediates the promoting effect of SF3B4 on EMT and ccRCC cell migration and invasion

**Fig. 7:**
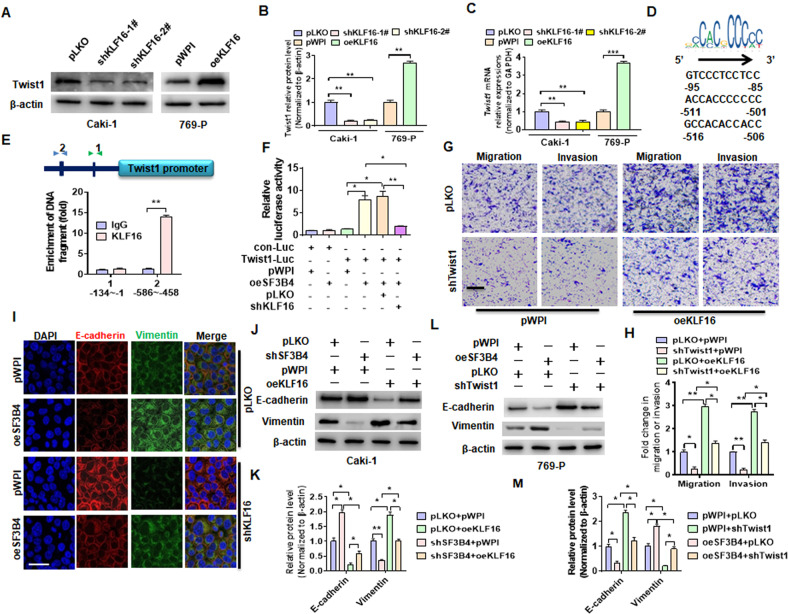
Upregulation of Twist1 by KLF16 mediates the promoting effect of SF3B4 on ccRCC progression. **A**–**C** Caki-1 and 769-P cells were transfected with indicated constructs, and then western blot and RT-qPCR detected Twist1 expression. **D** The potential KLF16-binding motif on the proximal promoter of Twist1 gene. **E** ChIP-PCR was used to verify the binding site of KLF16 on the Twist1 promoter. **F** Dual-luciferase reporter gene examined the role of SF3B4 and KLF16 in regulating Twist1 promoter activity by co-transfection with indicated constructs. **G**, **H** Caki-1 cells were transfected with shTwist1 and oeKLF16, alone or together, and then transwell assay was used to examine cell migration and invasion. Scale bar = 50 μm. **I** The expression of E-cadherin and vimentin were detected by double immunofluorescence staining in cells transfected with indicated constructs. Scale bar = 20 μm. **J**, **K** Caki-1 cells were transfected with shSF3B4 and oeKLF16, alone or together, and then the expression of EMT-related genes was detected by western blot. **L**, **M** 769-P cells were transfected with oeSF3B4 and shTwist1, alone or together, and then the expression of EMT-related genes was detected by western blot. All data are expressed as the mean ± SEM of three independent experiments. *P < 0.05, **P < 0.01, ***P < 0.001 vs. their corresponding controls.

Next, we explored whether KLF16 mediates the inducing effect of SF3B4 on Twist1 expression. Both gain- and loss-of-function studies demonstrated that the depletion of KLF16 in Caki-1 cells reduced, whereas its overexpression in 769-P cells increased Twist1 mRNA and protein expression (Fig. 7A–C). Considering that KLF16 is a transcription factor associated with tumor, we hypothesized that KLF16 might regulate Twist1 expression transcriptionally. Bioinformatically, we found three potential KLF16 binding sites on the Twist1 promoter (Fig. 7D). ChIP-PCR revealed that KLF16 was recruited to the two adjacent motifs between the −586 and −458 regions (Fig. 7E). The dual-luciferase reporter gene assay showed that depletion of KLF16 abolished SF3B4 overexpression-upregulated the activity of the Twist1 promoter, indicating that KLF16 mediates SF3B4 upregulation of Twist1 expression (Fig. 7F). Additionally, transwell assays showed that overexpression of KLF16 in Caki-1 cells significantly promoted cell migration and invasion, whereas depletion of Twist1 greatly counteracted the promoting effect of KLF16 overexpression on the migration and invasion (Fig. 7G, H). Further, we investigated whether KLF16 participates in SF3B4-induced EMT. Immunofluorescence staining showed that overexpression of SF3B4 in 769-P cells decreased E-cadherin but increased vimentin expression, which was almost completely reversed by simultaneously transfecting with shKLF16 (Fig. 7I). Otherwise, Western blot analysis revealed that overexpression of KLF16 in Caki-1 cells abrogated the inhibitory effect of shSF3B4 transfection on vimentin expression, with an opposite change in E-cadherin expression (Fig. 7J, K). In parallel, the deletion of Twist1 in 769-P cells blocked the promoting effect of SF3B4 overexpression on vimentin (Fig. 7L, M). Taken together, these results showed that the upregulation of Twist1 by KLF16 mediates the promoting effect of SF3B4 on EMT and ccRCC progression.

### SF3B4-KLF16-Twist1 axis promotes progression of ccRCC in vivo

**Fig. 8:**
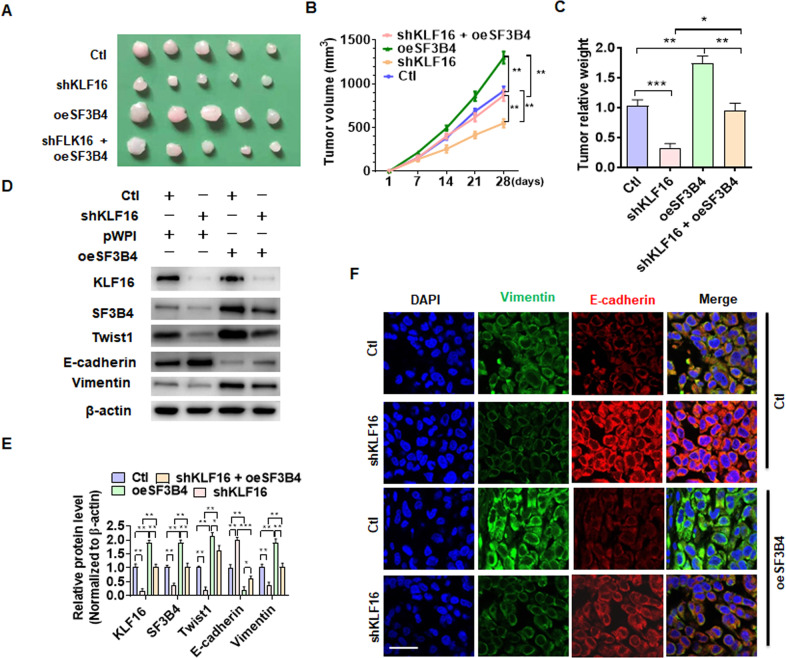
SF3B4-KLF16-Twist1 axis promotes the progression of ccRCC in vivo. **A** Caki-1 cells, engineered to stably knock down KLF16 or stably overexpress SF3B4, separately or together, were implanted into nude mice to establish xenograft tumors. The tumor sizes in each group were presented after 28 days. **B** Tumor volumes were measured by direct measurement with calipers. **C** Xenograft tumor wet weight was determined after the resection of tumors. **D** The expression of SF3B4, KLF16, Twist1, E-cadherin, and vimentin was examined in xenograft tumors by western blotting. **E** Quantitative analysis of **B**. **F** Double immunofluorescence staining of vimentin and E-cadherin in xenograft tumors. Scale bar = 20 μm. All data are expressed as the mean ± SEM of three independent experiments. *P < 0.05, **P < 0.01, ***P < 0.001 vs. their corresponding controls.

**Fig. 9:**
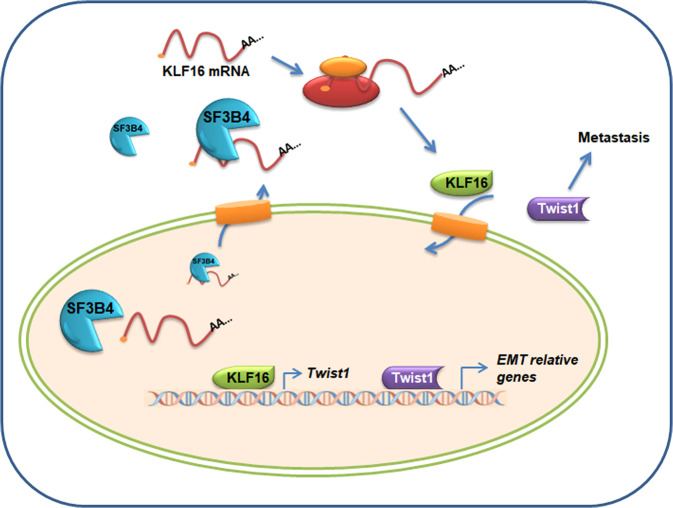
Proposed model for SF3B4-KLF16-Twist1 regulation of pRCC progression.

To determine whether the SF3B4-KLF16-Twist1 pathway is of pathophysiological relevance, we established a xenograft tumor model by implanting Caki-1 cells stably knocking down KLF16 and the cells stably overexpressing SF3B4, separately, or together into nude mice. As shown in Fig. 8A, the tumor volumes were obviously smaller in mice implanted with KLF16-depleted cells than in mice implanted with control cells. Reversely, stable overexpression of SF3B4 in implanted cells facilitated tumor growth. More importantly, stable knockdown of KLF16 largely attenuated the promoting effect of SF3B4 overexpression on tumor growth. The tumor volumes and wet weight showed the same results (Fig. 8B, C). Next, we detected epithelial and EMT marker gene expression in xenograft tumor tissues, and found that depletion of KLF16 in implanted cells dramatically reduced the expression of KLF16, Twist1, and mesenchymal markers vimentin, with an increase in the epithelial marker E-cadherin. As expected, SF3B4 overexpression substantially reversed the inhibitory effect of KLF16 depletion on EMT (Fig. 8D, E). Immunofluorescence staining of the xenograft tumor tissues yielded the same results (Fig. 8F). Collectively, these data provide a novel insight into the functional roles of SF3B4-KLF16-Twist1 axis in the development and progression of ccRCC, and manipulating this pathway may be a novel therapeutic target for the treatment of ccRCC (Fig. 9).

## Discussion

Distant metastasis of cancer cells is the most common cause of death from malignant tumors [36]. Under the action of different molecular mechanisms, tumor cells are able to infiltrate surrounding tissues, invade blood vessels and leave blood flow in different locations. These tumor cells often undergo a series of changes before they acquire the ability to metastasize, such as epithelial-mesenchymal transition, tumor cell interaction with extracellular matrix components, and angiogenesis [37]. Among them, EMT is one of the most important ways to obtain transfer ability. The EMT process is caused by epithelial cells losing the ability to adhere to neighboring cells and extracellular matrix proteins and acquiring a mesenchymal phenotype [30]. E-cadherin and ZO-1 are the key epithelial marker that enables cells to maintain an epithelial phenotype and are responsible for adherent junctions, while β-catenin and vimentin are mesenchymal markers required for cell migration [38]. The EMT process involves the downregulation of E-cadherin and upregulation of β-catenin and vimentin, resulting in decreased intercellular adhesion and increased cell migration [39]. The EMT process is regulated by diverse gene expression and complex factor networks, including cytokines, growth factors, signaling pathways, transcription factors, and the tumor microenvironment [40]. For example, EMT-related transcription factors such as Snail1, Snail2, ZEB1, ZEB2, TCF3, and KLF8 those bind to the E-cadherin promoter and repress its transcription, while factors such as Twist, Goosecoid, TCF4, The homeobox protein SIX1, and the forkhead box protein C2 (FOXC2) indirectly inhibit E-cadherin [17].

Twist1 is a member of the basic helix-loop-helix transcription factor family [41] and is involved in the regulation of cell differentiation, migration, proliferation, survival, and inflammatory responses. Its expression is required for normal embryogenesis, but is often upregulated during tumorigenesis and development [42]. Studies have shown that Twist1 binds to other bHLH dimers and regulates the transcription of EMT-related genes, and is involved in the metastasis of various cancers [43]. For example, Twist1 promotes the metastasis of osteosarcoma by up-regulating the expression of PCOLCE [44]. Twist1 promotes breast cancer invasion and metastasis by inhibiting the expression of Foxa1 [45]. MYC and Twist1 cooperate to drive metastasis by initiating crosstalk between hepatocellular carcinoma and innate immunity [46]. Twist1 regulates WDR5-Hottip-mediated Hoxa9 chromatin to promote prostate cancer metastasis [47]. TGF-β/Twist11/EMT regulates colorectal cancer cell migration and invasion mediated by long non-coding RNA TUG1 [48]. In ccRCC, Yin L. et al. [49] found that the hippo/TEAD1-Twist1 pathway is involved in SH3BGRL2-regulated ccRCC growth and metastasis. In addition, Twist1 expression in ccRCC was associated with a high Fuhrman grade, whereas its immune expression was more pronounced in advanced ccRCC [50]. Increased distribution of Twist1 in the cytoplasm was associated with higher-grade RCC and worse progression-free survival in ccRCC [51]. However, the upstream regulatory mechanism of Twist1 expression in ccRCC is still largely unknown. In the present study, we confirm Twist1 was upregulated in ccRCC tissues, and the higher expression of Twist1 usually with poor overall survival of patients. Importantly, the upregulation of Twist1 mediated SF3B4-promoted the cell migration and invasion of ccRCC. In addition, knockdown of SF3B4 in Caki-1 cells inhibited cell proliferation, while overexpression of SF3B4 in 769-P cells promoted cell growth (data not show). Novelty, we revealed that the upreguation of SF3B4 accelerated the mRNA export and facilitated the expression of transcription factor KLF16 which binding to promoter and promote Twist1 expression. The results demonstrated that SF3B4-KLF16-Twist1 axis plays a critical key role in ccRCC cell EMT and metastasis. Although our results show that the knockdown of KLF16 does not completely reverse the effect of SF3B4 overexpression. The most likely reason is the presence of redundant pathways involved in the progress of SF3B4 regulation of ccRCC. In the next step, we will also delve into other mechanisms of action of SF3B4 in ccRCC.

As the core subunit of the U2 spliceosome, the splicing factor SF3B4 not only plays a vital role in the splicing process, but also plays a key role in transcription, translation, and cell signal transduction, and participates in the regulation of cell cycle, cell differentiation and immune deficiency [52]. In recent years, more and more research has been conducted on SF3b4-related diseases such as Nagel syndrome and cancer [53]. Recently, Diao et al. [54] found that SF3B4 promotes ovarian cancer progression by modulating alternative splicing of RAD52 [14]. SF3B4 is also closely related to the growth of non-small cell lung cancer cells. All of the above indicate the protocarcinological role of SF3B4 in tumors. On the other hand, a large number of studies have also shown that KLF16, as a protocarcinofactor, is also involved in the occurrence and development of a variety of tumors, including ccRCC [27–29, 55]. However, the relationship between SF3B4 and KLF16 including ccRCC in tumors is unclear. A large number of studies have shown that SF3B4 as a splicing factor plays a regulatory role in the splicing of gene RNA. Although upregulation of SF3B4 may promote mRNA maturation and cytosolic transport by promoting RNA splicing. But current evidence suggests that gene splicing generally occurs within the nucleus, especially for the KLF16 gene. But our results revealed that (1) SF3B4 can bind to KLF16 RNA and is distributed in both the nucleus and cytoplasm. (2) Upregulation of SF3B4 expression further enhanced KLF16 mRNA cytoplasmic distribution, and their interactions were also enhanced. (3) Upregulation of SF3B4 expression does not affect the distribution of control RNA NEAT1 RNA in the cell. Therefore, we believe that SF3B4 upregulates KLF14 expression by promoting the nucleation of mRNA.

## Conclusion

In summary, our studies show that the upregulation of SF3B4 in ccRCC tissues leads to the formation of SF3B4-KLF16-Twist1 regulatory axis which promotes ccRCC progression by facilitating cell EMT and migration. As a component of the splicing factor as well as RNA binding protein, we discovered a novel function of SF3B4 promoted expression of KLF16 by facilitating mRNA export from nucleus. The upregulation of KLF16 binding to the promoter and elevated transcription of Twist1. Targeting this newly identified regulatory axis may provide therapeutic benefits against ccRCC metastasis.

## Supplementary information

Reproducibility checklist

Supplementary Figures

SUPPLEMENTAL MATERIAL

Supplementary table 1

Supplementary table 2

Supplementary table 3

Supplementary table 4

## Data Availability

The published article includes all data sets generated/analyzed for this study.
