# Supplementary material for: SF3B4 promotes Twist1 expression and clear cell renal cell carcinoma progression by facilitating the export of KLF 16 mRNA from the nucleus to the cytoplasm
Source: Cell Death Dis. 2023 Jan 13;14(1):26. doi: 10.1038/s41419-022-05534-w (PMC9839716; doi:10.1038/s41419-022-05534-w)
Supplement: Supplementary file 2 — Supplementary Figures [file 41419_2022_5534_MOESM2_ESM.docx]

**SF3B4 promotes Twist1 expression and clear cell renal cell carcinoma progression by facilitating the export of KLF 16 mRNA from the nucleus to the cytoplasm**

Zhan Yang ^1,3#^, Ya-Xuan Wang ^1#^, Jin-Kun Wen ^2^, Hai-Tao Gao ^1^, Zhen-Wei Han ^1^, Jin-Chun Qi ^1^, Jun-Fei Gu ^1^, Chen-Ming Zhao ^1^, Hong Zhang ^1^, Bei Shi ^1^, Dan-Dan Wang ^1^, Xiao-Lu Wang ^1^, Chang-Bao Qu ^1*^

^1^ Department of Urology, The Second Hospital of Hebei Medical University, 215 Heping W Rd, Shijiazhuang, 050000, China. ^2^ Department of Biochemistry and Molecular Biology, Ministry of Education of China, Hebei Medical University, No. 361 Zhongshan E Rd, Shijiazhuang, 050017, China. ^3^ Molecular Biology Laboratory, Talent and Academic Exchange Center, The Second Hospital of Hebei Medical University, Shijiazhang, China

**Supplementary materials**


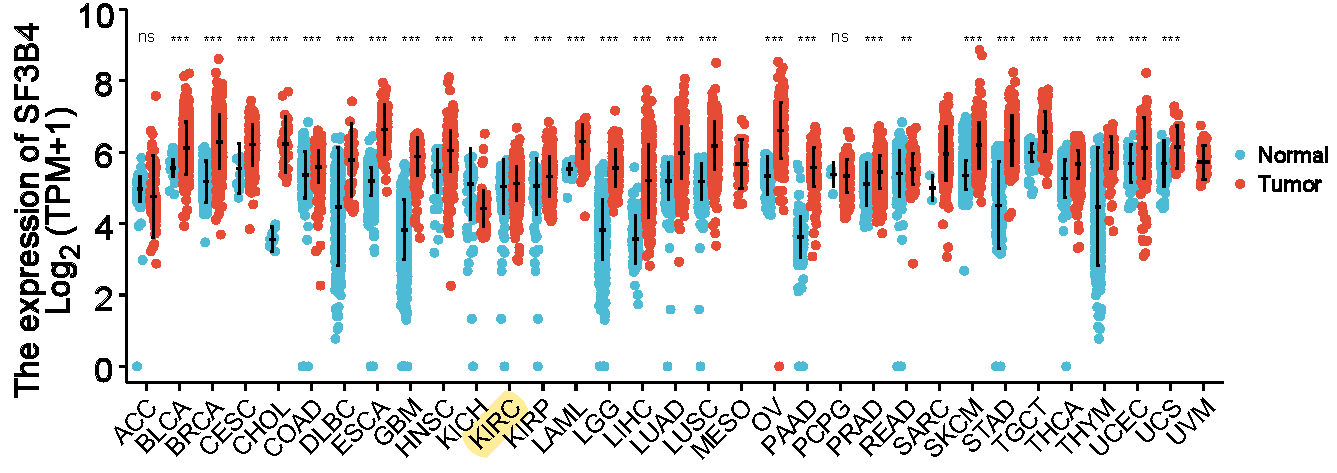


**Supplementary Fig. 1.** SF3B4 mRNA expression in different types of human cancers based on TCGA dataset.

**A**

**B**

**Supplementary Fig. 2. RT-qPCR detects SF3B4 expression in ccRCC cell lines.** **A,** Caki-1 cell was transfected with shSF3B4-1# or shSF3B4-2# or pLKO control vector and then RT-qPCR detected the SF3B4 mRNA expression. **B,** 769-P cell was transfected with oeSF3B4 or pWPI control vector, and then RT-qPCR detected the SF3B4 mRNA expression. **P<0.01, ***P<0.001 vs. indicated control.

**A**


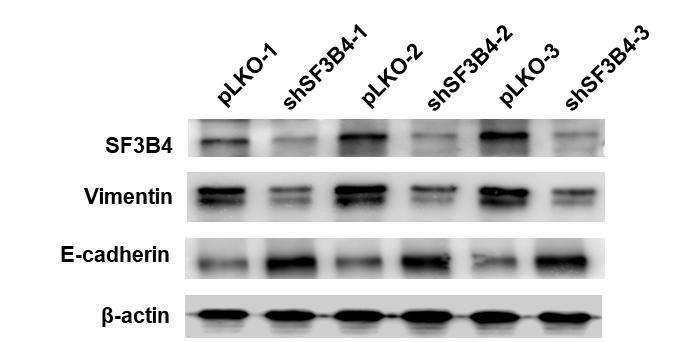


**B**


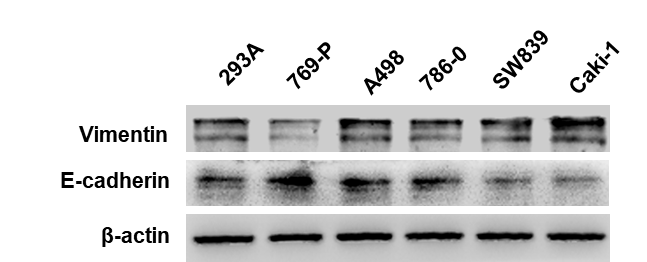


**Supplementary Fig. 3. Western blot was used to detect EMT relative gene expression. A,** Three batches of Caki-1 cells were transfected with shSF3B4 or pLKO control vector and then Western blot was used to examine SF3B4, Vimentin and E-cadherin protein expression. **B,** Western blot was used to examine Vimentin and E-cadherin protein expression in different cell lines.


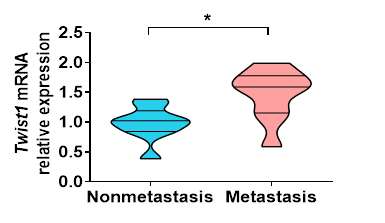


**Supplementary Fig. 4.** RT-qPCR was used to detected the Twist1 expression in nonmetastasis and metastasis tissues. *P<0.05 vs. nonmetastasis.


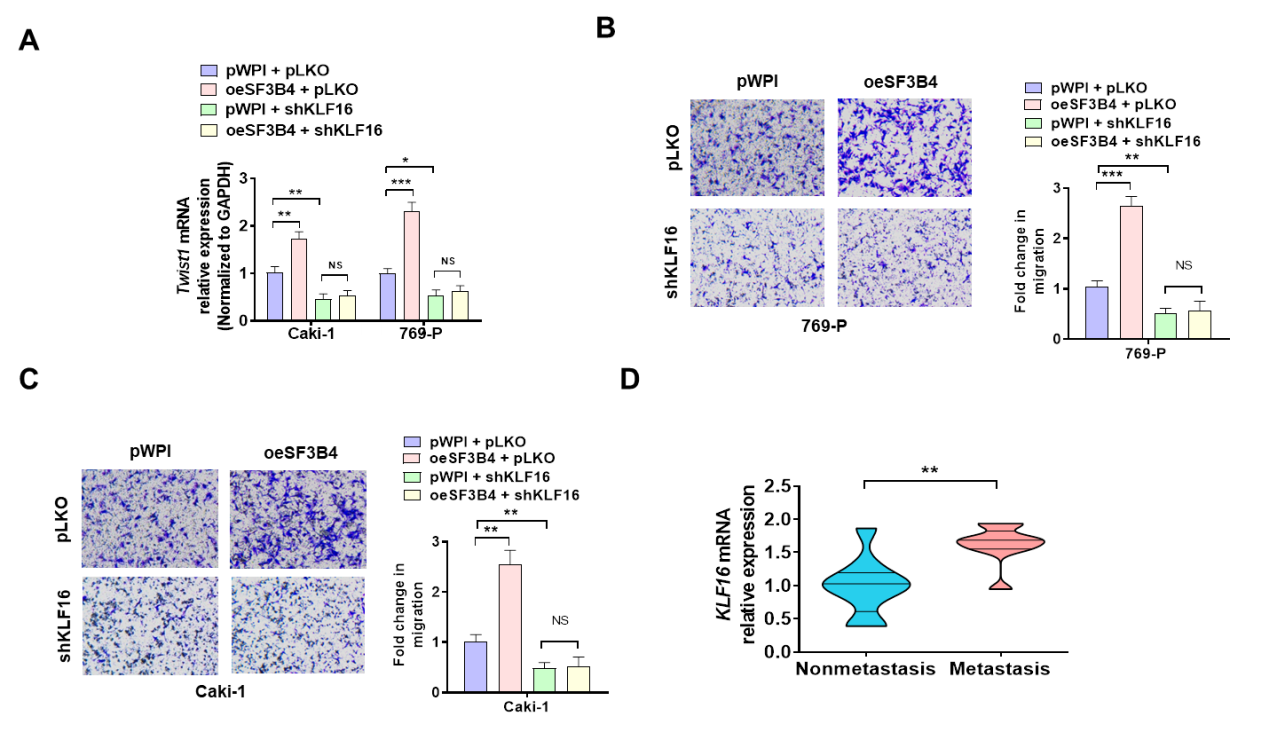


**Supplementary Fig. 5. KLF16 mediates SF3B4 upregulation of Twist1 expression in ccRCC cells. A,** Caki-1 and 769-P cells were transfected with oeSF3B4 or shKLF16, alone or both together, and then RT-qPCR detected Twist1 expression. **B and C,** Transwell assay was used to detect 769-P and Caki-1 cells migration transfection with oeSF3B4 or shKLF16 alone or together. **D,** RT-qPCR was used to detected the KLF16 expression in nonmetastasis and metastasis tissues. *P<0.05, **P<0.01,***P<0.001 vs. indicated control.


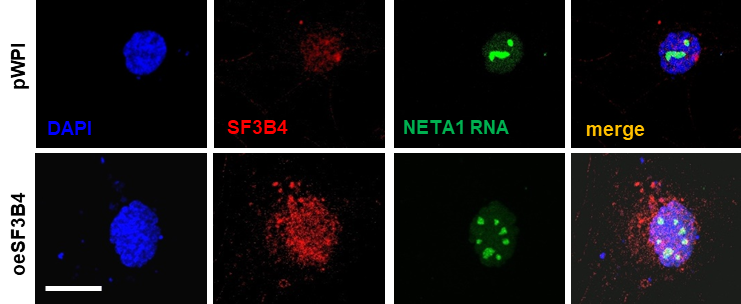


**Supplementary Fig. 6.** Caki-1cells were transfected with oeSF3B4or pWPI, and the expression of SF3B4 protein and NEAT1 RNA as well as their distribution in the nucleus and cytoplasm were detected by immunofluorescence combined with FISH. SF3B4 antibody (red) was used to detect SF3B4 protein, while the FITC-probe (green) was used to detect NEAT1 RNA.
