# Supplementary material for: SF3B4 promotes Twist1 expression and clear cell renal cell carcinoma progression by facilitating the export of KLF 16 mRNA from the nucleus to the cytoplasm
Source: Cell Death Dis. 2023 Jan 13;14(1):26. doi: 10.1038/s41419-022-05534-w (PMC9839716; doi:10.1038/s41419-022-05534-w)
Supplement: Supplementary file 3 — SUPPLEMENTAL MATERIAL [file 41419_2022_5534_MOESM3_ESM.docx]

Supplementary materials


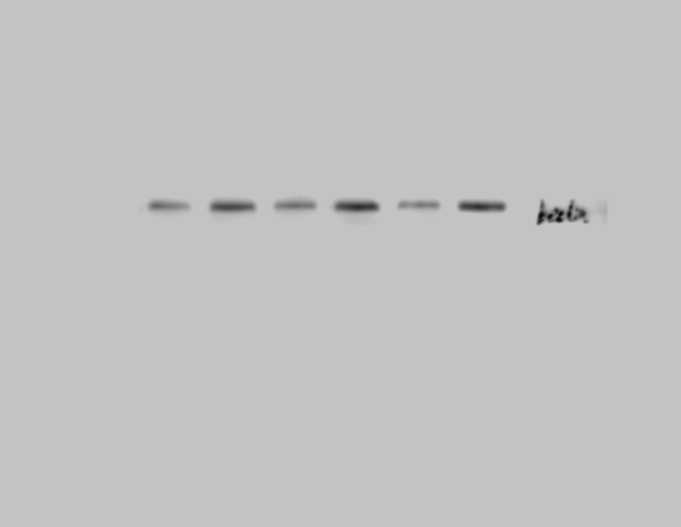


**kDa**

**55**

**43**

**SF3B4**


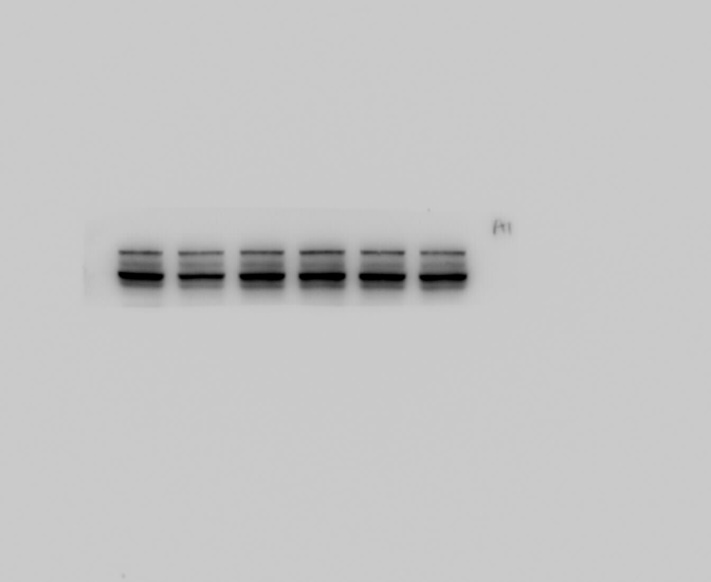


**β-actin**

**kDa**

**43**

**34**

**Supplementary Figure 7.** The original immunoblots of Figure 1C.


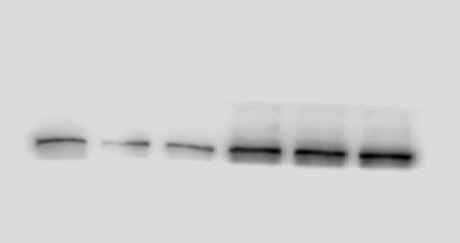


**kDa**

**55**

**43**

**SF3B4**


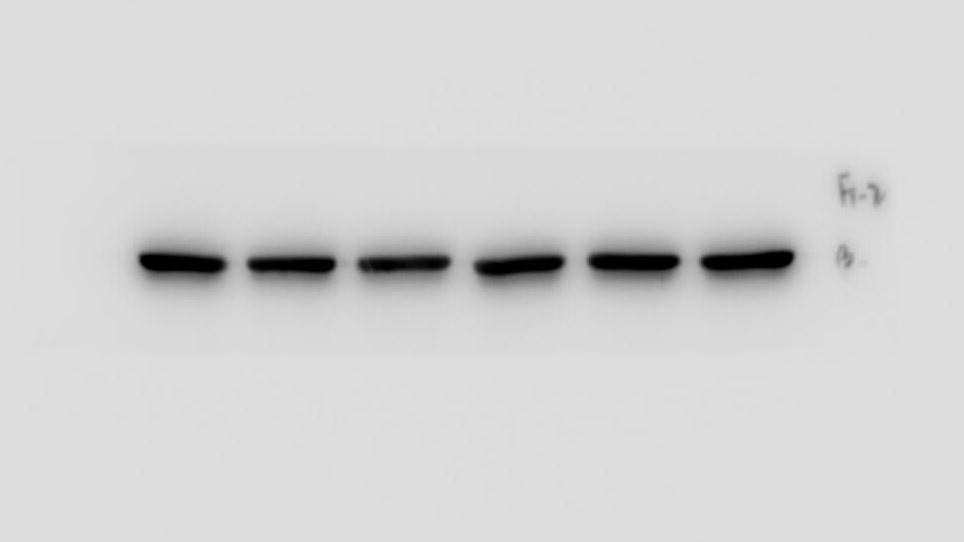


**β-actin**

**kDa**

**43**

**34**

**Supplementary Figure 8.** The original immunoblots of Figure 2B.


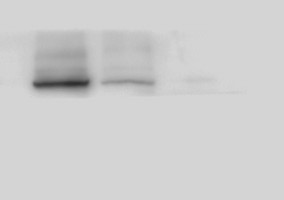


**SF3B4**

**kDa**

**55**

**43**


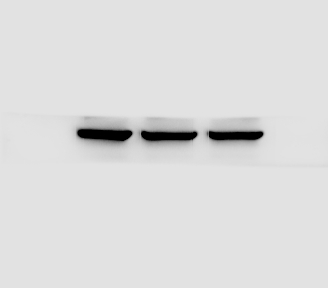


**β-actin**

**kDa**

**43**

**34**

**Supplementary Figure 9.** The original immunoblots of Figure 2D.


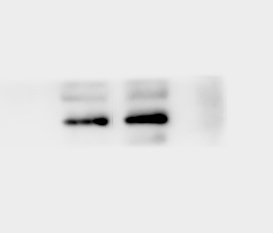


**SF3B4**

**kDa**

**55**

**43**


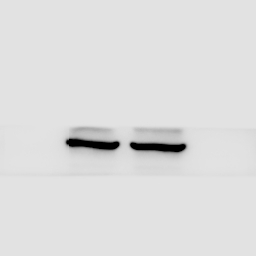


**β-actin**

**kDa**

**43**

**34**

**Supplementary Figure 10.** The original immunoblots of Figure 2F.

**E-cadherin**

**kDa**

**115**

**75**


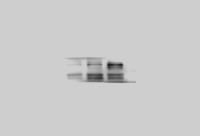


**E-cadherin**

**kDa**

**115**

**75**


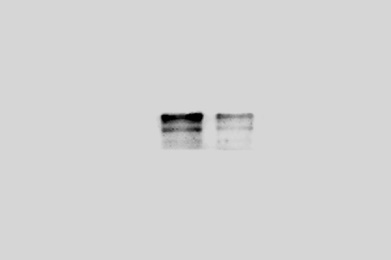


**Vimentin**

**55**

**43**

**kDa**


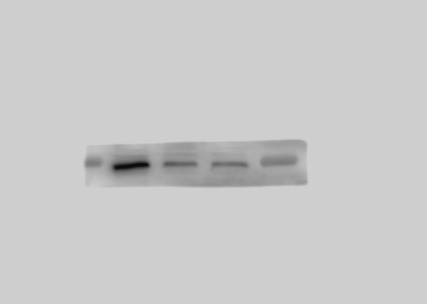


**Vimentin**

**kDa**

**55**

**43**


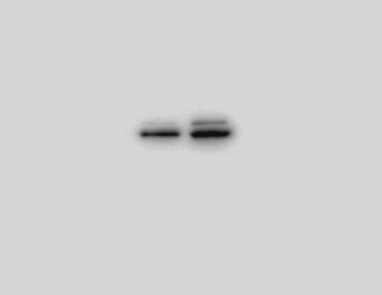


**25**

**17**

**kDa**


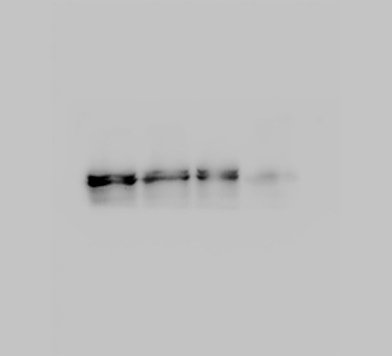


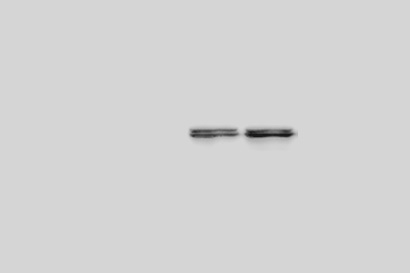


**kDa**

**25**

**17**

**Twist1**

**Twist1**


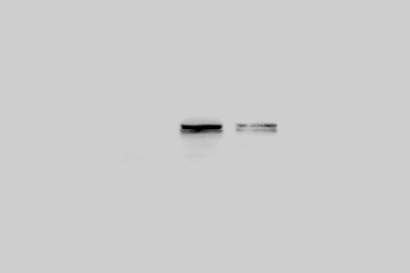


**kDa**

**180**

**180**

**kDa**


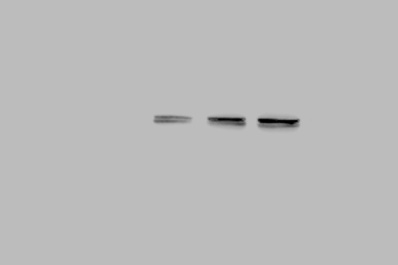


**ZO-1**

**ZO-1**


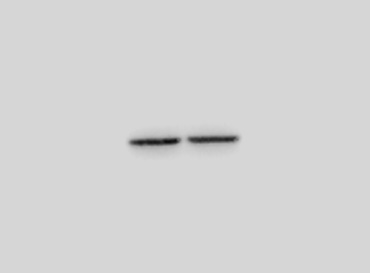


**43**

**34**

**kDa**


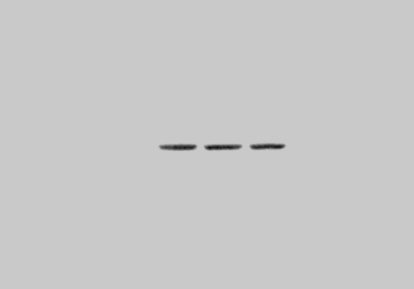


**kDa**

**43**

**34**

**β-actin**

**β-actin**

**Supplementary Figure 11.** The original immunoblots of Figure 3D.


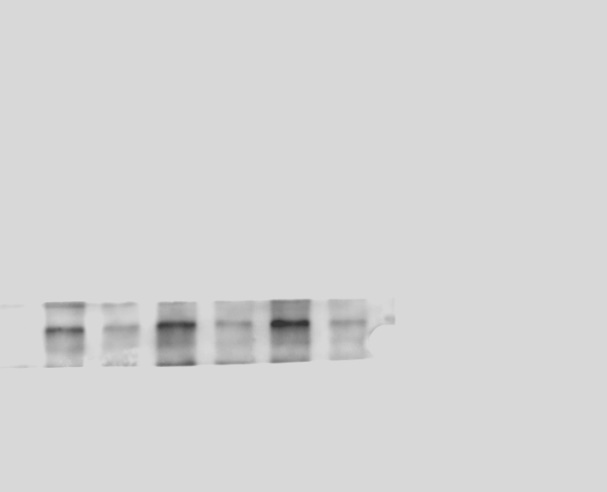


**SF3B4**

**kDa**

**55**

**43**


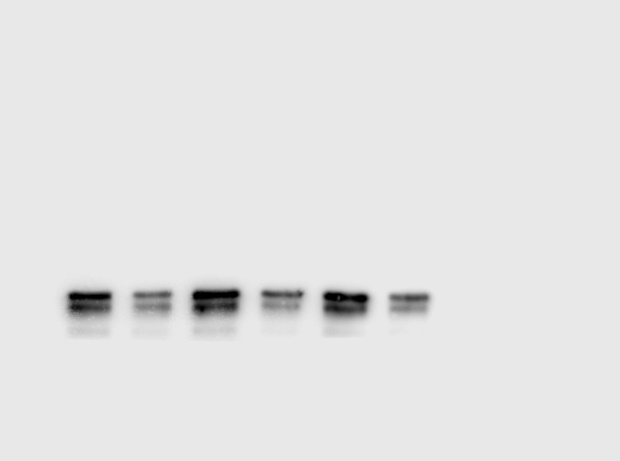


**Vimentin**

**kDa**

**55**

**43**


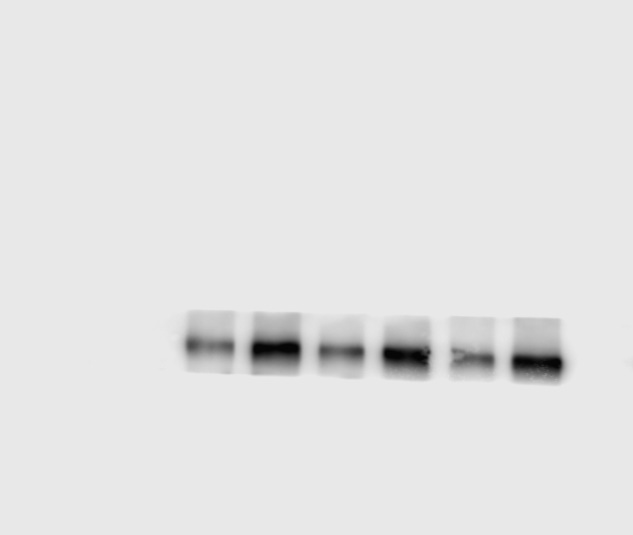


**E-cadherin**

**kDa**

**115**

**75**


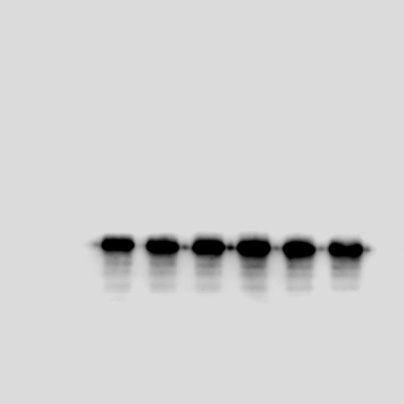


**β-actin**

**kDa**

**43**

**34**

**Supplementary Figure 12.** The original immunoblots of **Supplementary Figure 3A**.


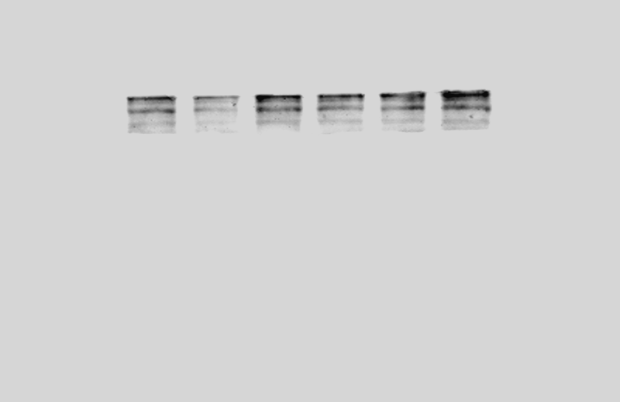


**Vimentin**

**kDa**

**55**

**43**

**kDa**

**115**

**75**


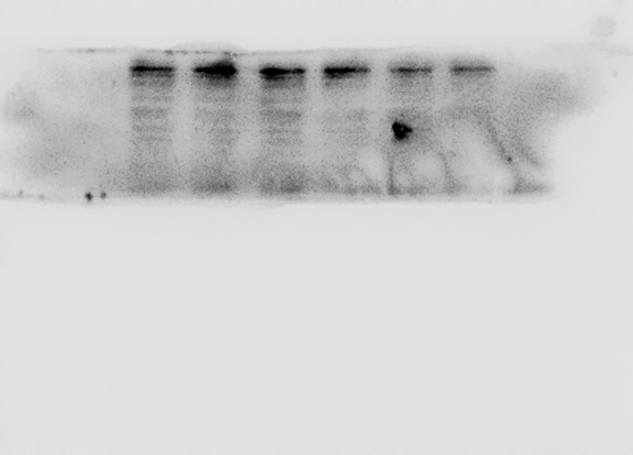


**E-cadherin**


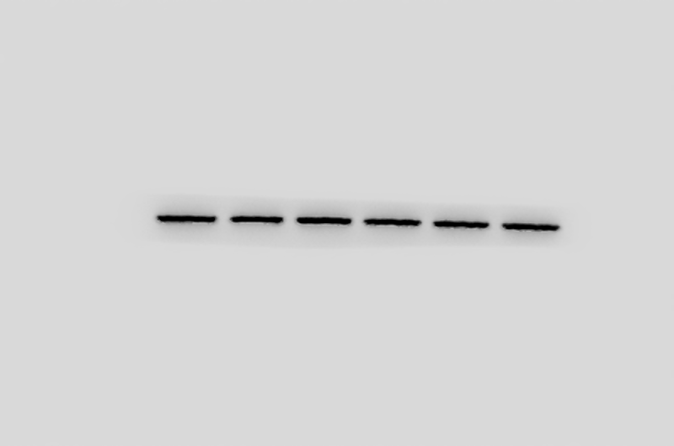


**β-actin**

**kDa**

**43**

**34**

**Supplementary Figure 13.** The original immunoblots of **Supplementary Figure 3B**.


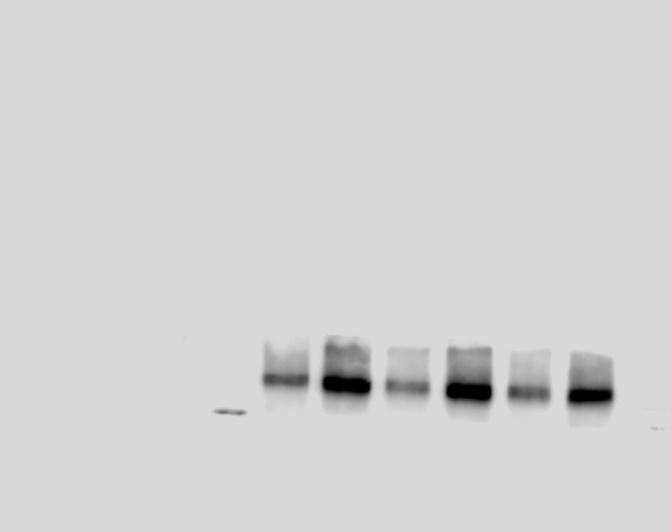


**Twist1**

**kDa**

**25**

**17**


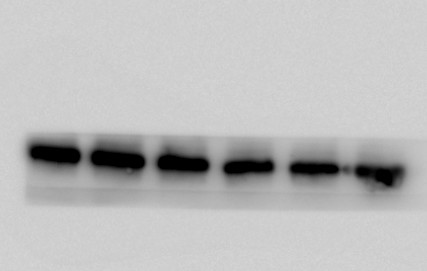


**β-actin**

**kDa**

**43**

**34**

**Supplementary Figure 14.** The original immunoblots of **Supplementary Figure 4F**.


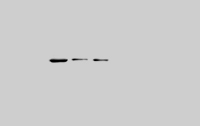


**kDa**

**34**

**25**


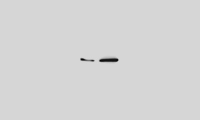


**kDa**

**34**

**25**

**KLF16**

**KLF16**

**β-actin**

**kDa**

**43**

**34**


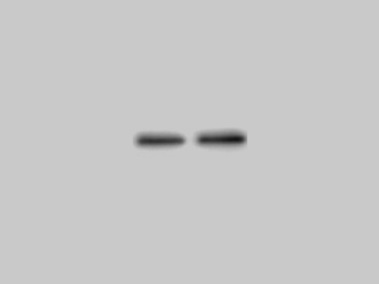


**43**

**34**

**kDa**


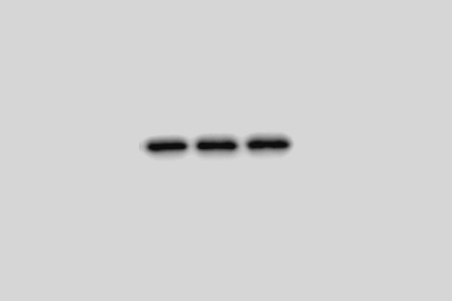


**β-actin**

**Supplementary Figure 15.** The original immunoblots of Figure 5C.


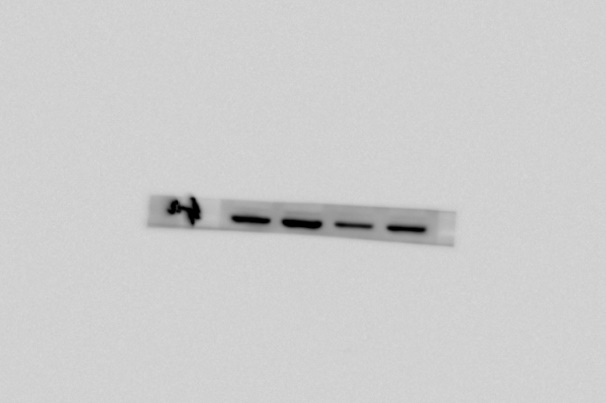


**KLF16**

**kDa**

**34**

**25**


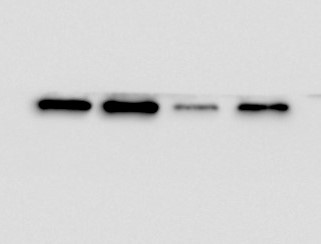


**Twist1**

**kDa**

**25**

**17**


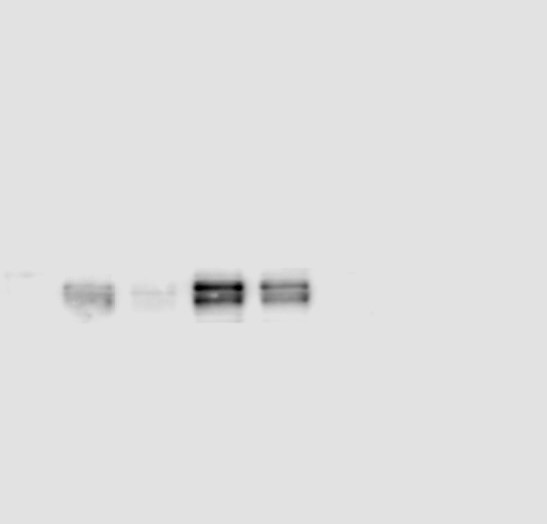


**E-cadherin**

**kDa**

**115**

**75**


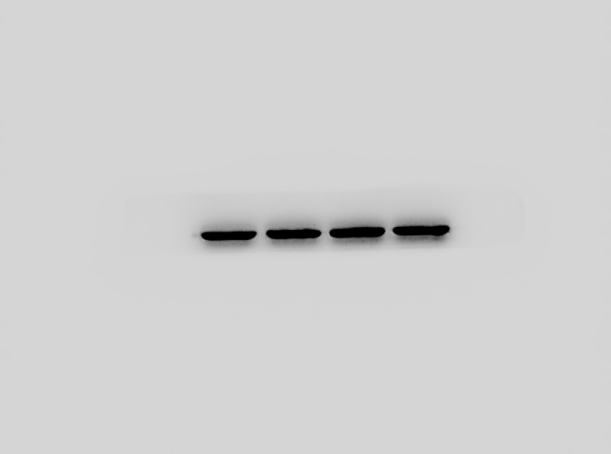


**β-actin**

**kDa**

**43**

**34**

**Supplementary Figure 16.** The original immunoblots of **Supplementary Figure 5H**.


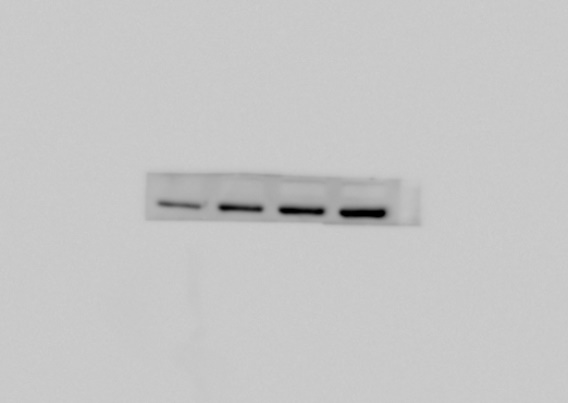


**KLF16**

**kDa**

**34**

**25**


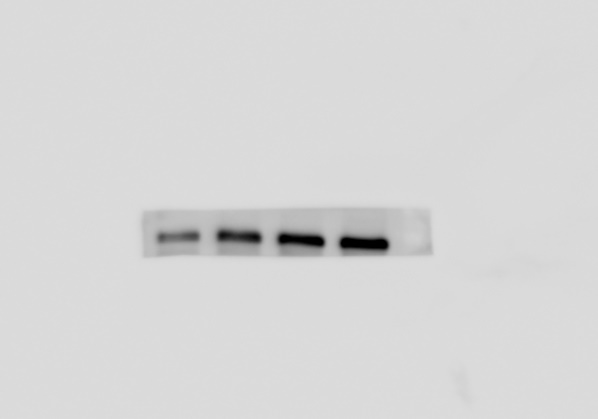


**kDa**

**25**

**17**

**Twist1**


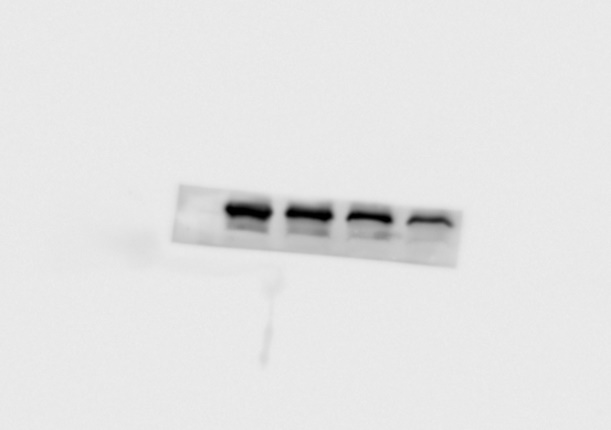


**E-cadherin**

**kDa**

**115**

**75**


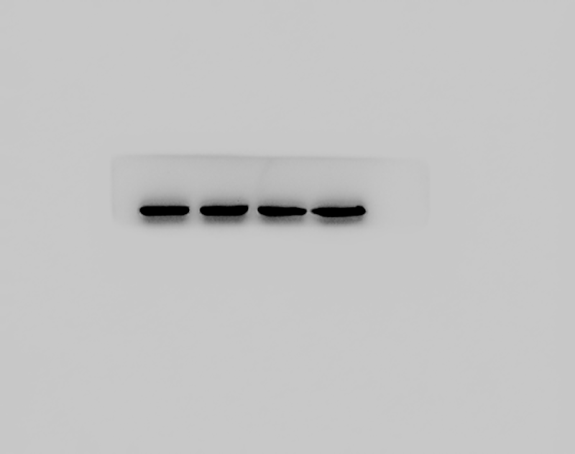


**β-actin**

**kDa**

**43**

**34**

**Supplementary Figure 17.** The original immunoblots of **Supplementary Figure 5J**.


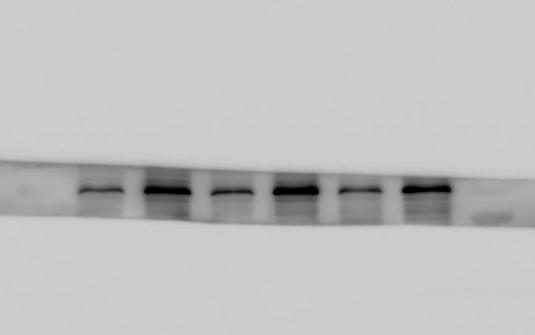


**kDa**

**34**

**25**

**KLF16**


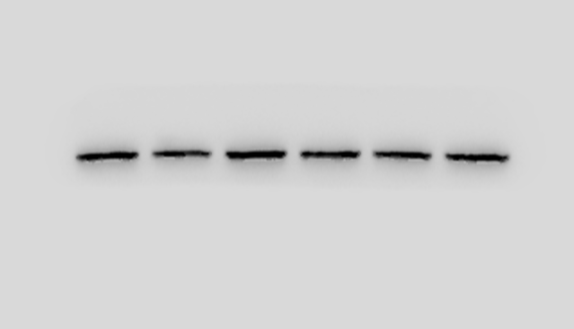


**β-actin**

**kDa**

**43**

**34**

**Supplementary Figure 18.** The original immunoblots of Supplementary Figure 5M.


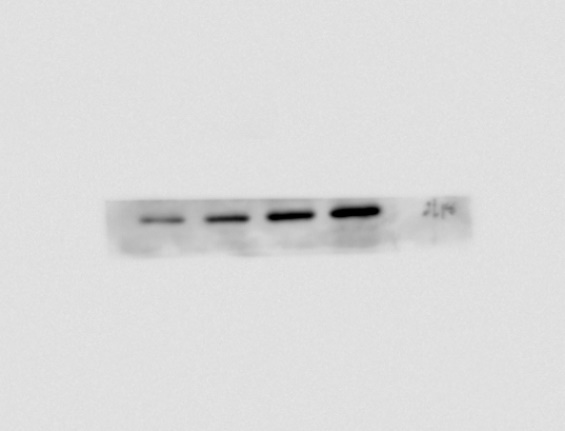


**KLF16**

**kDa**

**34**

**25**


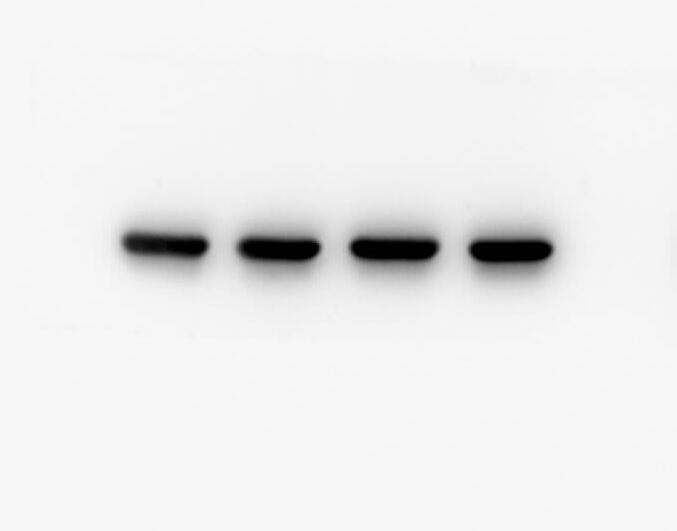


**β-actin**

**kDa**

**43**

**34**


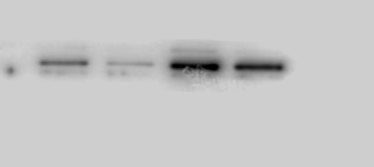


**KLF16**

**kDa**

**34**

**25**


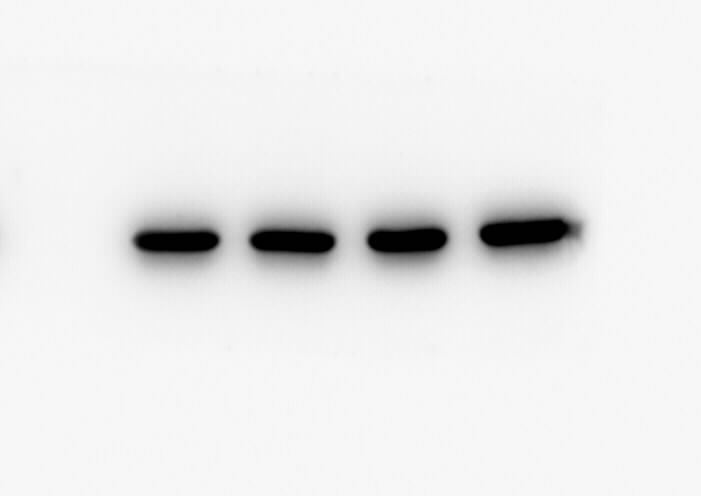


**β-actin**

**kDa**

**43**

**34**

**Supplementary Figure 19.** The original immunoblots of Supplementary Figure 6A.


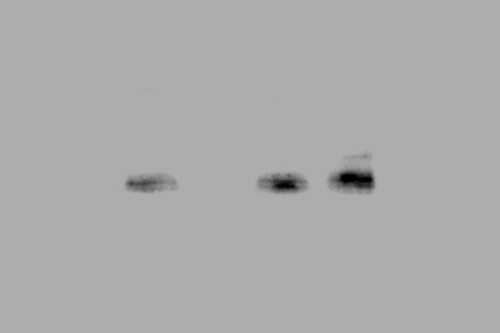


**kDa**

**55**

**44**

**SF3B4**


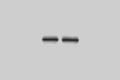


**kDa**

**43**

**34**

**β-actin**

**Supplementary Figure 20.** The original immunoblots of Figure 6D.


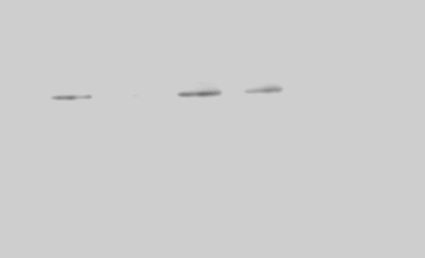


**SF3B4**

**kDa**

**55**

**44**

**Supplementary Figure 21.** The original immunoblots of Figure 6F.


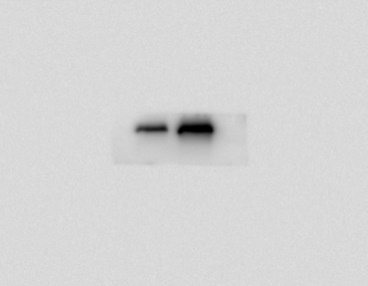

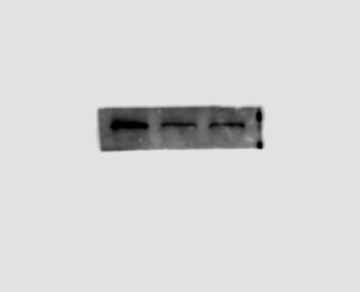


**kDa**

**25**

**17**

**kDa**

**25**

**17**

**Twist1**

**Twist1**

**β-actin**

**kDa**

**43**

**34**


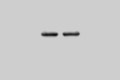


**43**

**34**

**kDa**


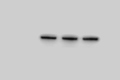


**β-actin**

**Supplementary Figure 22.** The original immunoblots of Figure 7A.


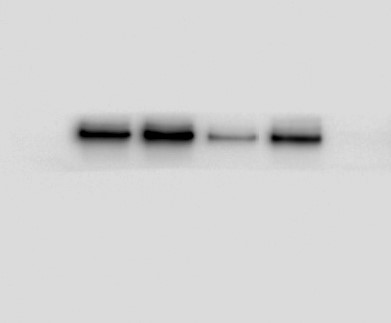


**E-cadherin**

**kDa**

**115**

**75**


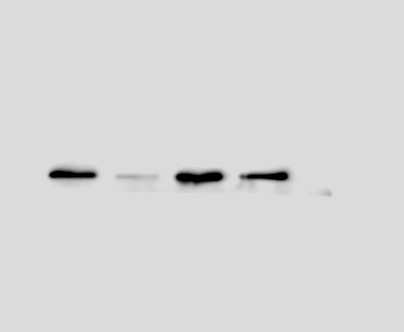


**Vimentin**

**kDa**

**55**

**43**


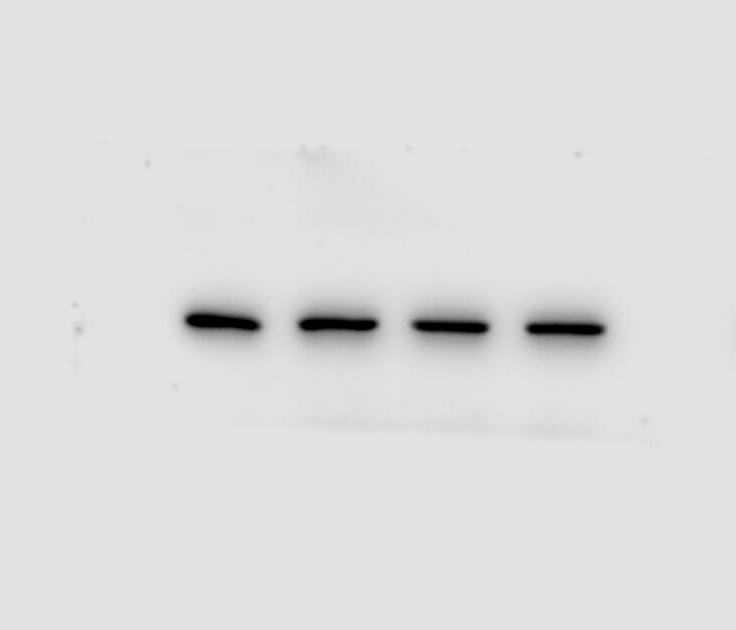


**β-actin**

**kDa**

**43**

**34**

**Supplementary Figure 23.** The original immunoblots of **Supplementary Figure 7J**.


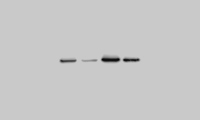


**E-cadherin**

**kDa**

**115**

**75**


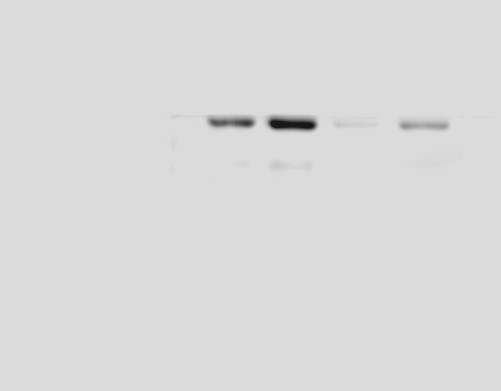


**Vimentin**

**kDa**

**55**

**43**


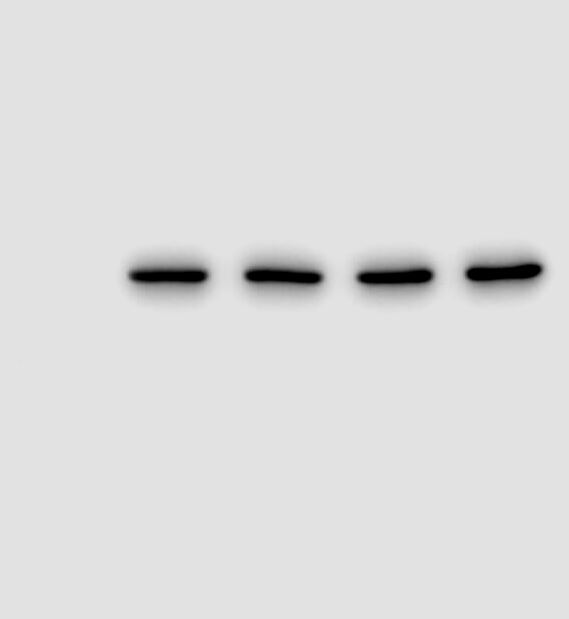


**β-actin**

**kDa**

**43**

**34**

**Supplementary Figure 24.** The original immunoblots of **Supplementary Figure 7L**.


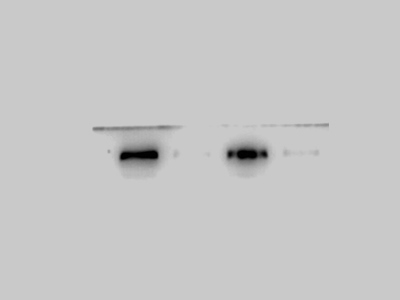


**KLF16**

**kDa**

**34**

**25**


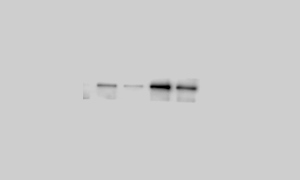


**kDa**

**55**

**43**

**SF3B4**


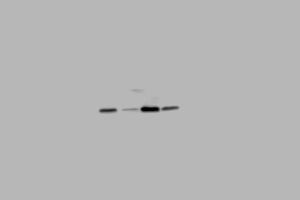


**kDa**

**43**

**34**

**Twist1**


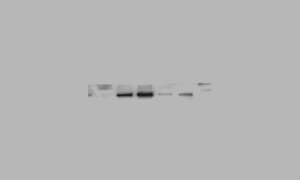


**kDa**

**115**

**75**

**E-cadherin**


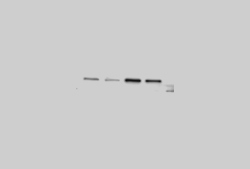


**Vimentin**

**kDa**

**55**

**43**


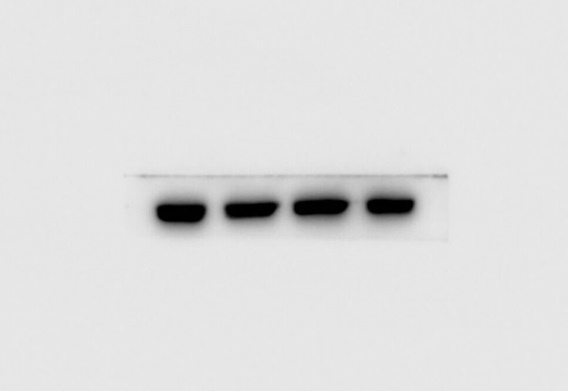


**kDa**

**43**

**34**

**β-actin**

**Supplementary Figure 25.** The original immunoblots of **Supplementary Figure 7L**.
