# Supplementary material for: SF3B4 promotes Twist1 expression and clear cell renal cell carcinoma progression by facilitating the export of KLF 16 mRNA from the nucleus to the cytoplasm
Source: Cell Death Dis. 2023 Jan 13;14(1):26. doi: 10.1038/s41419-022-05534-w (PMC9839716; doi:10.1038/s41419-022-05534-w)
Supplement: Supplementary file 4 — Supplementary table 1 [file 41419_2022_5534_MOESM4_ESM.docx]

**Supplementary table**  primers used in the study

| Name | Sequcence 5’ to 3’ |
| --- | --- |
| SF3B4-F1 | GGGAAGCCAATACGGGTGAA |
| SF3B4-R1 | AAGCCCAGACCCCAATGATG |
| Snail1-F | CGAGTGGTTCTTCTGCGCTA |
| Snail1-R | GGGCTGCTGGAAGGTAAACT |
| Snail2-F | AACAGTATGTGCCTTGGGGG |
| Snail2-R | AAAAGGCACTTGGAAGGGGT |
| ZEB1-F | GATGACCTGCCAACAGACCA |
| ZEB1-R | GTGTCATCCTCCCAGCAGTT |
| ZEB2-F | AGCCTCTGTAGATGGTCCAGT |
| ZEB2-R | GGTCAGCAGTTGGGCAAAAG |
| MMP1-F | AGAAAGAAGACAAAGGCAAGTTGA |
| MMP1-R | CTCTTGGCAAATCTGGCGTG |
| TCF3-F | TCCCTGGAGGAGAAAGACCT |
| TCF3-R | GGCCTCGTTAATATCCCGCA |
| Twist1-F | CTTCTCGGTCTGGAGGATGG |
| Twist1-R | GCACGACCTCTTGAGAATGC |
| GRHL2-F | CTTCACCTGCACAGACTTGA |
| GRHL2-R | GCGCGTGTGATCCACTTACT |
| SIX1-F | TGGTTTAAGAACCGGAGGCA |
| SIX1-R | AGTCCCTCCCCACTTAGGAC |
| FOXCUT-F | GCCGGGCACCAAAGTTAAAG |
| FOXCUT-R | CGACCTTGGGCAGATACTCC |
| KLF16-F | CGCCAAAGCCTACTACAAGT |
| KLF16-R | CCTGCCAGTCACAAGCAAAAG |
| GAPDH-F: | AATGGGCAGCCGTTAGGAAA |
| GAPDH-R: | GCGCCCAATACGACCAAATC |
| Twist1-pro-F1 | GGACTGGAAAGCGGAAACTTTCC |
| Twist1-pro-R1 | GGGCGAGAGCTGCAGACTTGG |
| Twist1-pro-F2 | GGGTTCGTCTACCTGACCATTGG |
| Twist1-pro-R2 | GAGCCCTCTAGGTCCGTGG |
| si-EZH2-F | GCGGGACGAAGAAUAAUCAUU |
| si-EZH2-R | UGAUUAUUCUUCGUCCCGCUU |
| si-TAF6-F | CCGGGAGCUUUACUUCUAUUU |
| si-TAF6-R | AUAGAAGUAAAGCUCCCGGUU |
| si-TFAP4-F | CUAUGGAGUAUUUCAUGGUUU |
| si-TFAP4-R | ACCAUGAAAUACUCCAUAGUU |
| si-KLF16-F | GCCAAAGCCUACUACAAGUUU |
| si-KLF16-R | ACUUGUAGUAGGCUUUGGCUU |
| si-TBX5-F | GGCACGGAAAUGAUCAUAAUU |
| si-TBX5-R | UUAUGAUCAUUUCCGUGCCUU |
| si-ATF1-F | GGCAGCCACAGUUGAUUAUUU |
| si-ATF1-R | AUAAUCAACUGUGGCUGCCUU |
| si-SP1-F | GUGCAAACCAACAGAUUAUUU |
| si-SP1-R | AUAAUCUGUUGGUUUGCACUU |
| si-MITF-F | UGGACUAUAUCCGAAAGUUU |
| si-MITF-R | ACUUUCGGAUAUAGUCCACUU |
| si-MAFK-F | CUAAUCCCAAACCGAAUAAUU |
| si-MAFK-R | UUAUUCGGUUUGGGAUUAGUU |
| si-TEAD4-F | CGCCAAAUCUAUGACAAAUUU |
| si-TEAD4-R | AUUUGUCAUAGAUUUGGCGUU |
| si-PRR7-F | GAGGAUAAUAAAGGUGUGUUU |
| si-PRR7-R | ACACACCUUUAUUAUCCUCUU |
|  |  |
|  |  |
|  |  |
